# Bonding Interface and Repairability of 3D-Printed Intraoral Splints: Shear Bond Strength to Current Polymers, with and without Ageing

**DOI:** 10.3390/ma14143935

**Published:** 2021-07-14

**Authors:** Ebru Kuscu, Andrea Klink, Sebastian Spintzyk, Pablo Kraemer Fernandez, Fabian Huettig

**Affiliations:** 1 Department of Prosthodontics, University Clinic of Dentistry, Oral Medicine, and Maxillofacial Surgery with Dental School, Tuebingen University Hospital, Osianderstr. 2–8, 72076 Tübingen, Germany; Andrea.Klink@med.uni-tuebingen.de (A.K.); pablo.kraemer-fernandez@med.uni-tuebingen.de (P.K.F.); fabian.huettig@med.uni-tuebingen.de (F.H.); 2 Section Medical Materials Science and Technology, Tuebingen University Hospital, Osianderstr. 2–8, 72076 Tübingen, Germany; sebastian.spintzyk@med.uni-tuebingen.de

**Keywords:** mechanical properties, surface characteristics, additive manufacturing, failure mode, light curing resin

## Abstract

This in-vitro study investigates the bonding interfaces reached by the conditioning of a splint material additively manufactured by digital light processing (AM base) as well as the shear bond strength (SBS) of resins bonded to these surfaces (repair material). Therefore, the AM base was either stored in dry for 12 h or wet environment for 14 days to simulate ageing by intraoral wear. The dry and wet group was bonded after physical and/or chemical conditioning to cylinders made from polymethylmethacrylate or four novel polymers allowing splint modifications. Blasted and methylmethacrylate (MMA)-conditioned Polymethylmethacrylate (PMMA) bonded to PMMA acted as the gold standard. The surface profiles revealed highest differences of Ra towards the gold standard in AM base conditioned with other than MMA after sandblasting. The adhesively bonded repair materials of the wet AM base were further aged in wet environment for 14 days. The SBS of the gold standard (25.2 MPa and 25.6 MPa) was only reached by PMMA bonded to blasted and MMA-conditioned AM base after dry (22.7 MPa) and non-conditioned after wet storage (23 MPa). Four repair materials failed to reach the threshold of 5 MPa after dry storage and three after wet storage, respectively. Non-conditioned AM base revealed the highest risk for adhesive fractures when using other resins than PMMA.

## 1. Introduction

Intraoral splints are medical devices that facilitate the treatment of bruxism, craniomandibular disorders [1,2,3,4], jaw pain, or support surgical approaches [5,6] (corrective osteotomy of the jaws). In common dental practice, intraoral splints act most prevalently as bite guards [7,8]. Polymethylmethacrylates (PMMA) are the gold standard of conventional manufacturing [9], either in terms of autopolymerizing methacrylate monomer and polymer in powder liquid technique partially combined with vacuum molded polyethylen frameworks [10,11,12,13] or in terms of CAD/CAM-milled from industrial polymerized blanks.

Today, additive manufacturing allows a direct manufacturing of computer-aided designed splints based on intra- or extraoral jaw scans [14,15,16]. Therefore, light-curing resins containing (meth) acrylates as well as initiators for the photopolymerization and fillers are provided for digital light processing (DLP) technology or stereolithography (SLA) [17,18,19,20].

Clinically, intraoral splints must be modified occasionally in terms of material addition directly after manufacturing (in the dental lab) or after a period of intraoral service (chairside or in the dental lab). This measure is necessary to obtain further/other functional areas on the occlusal surface or to repair minor destructions due to the bite forces and other causes which may lead to appliance fractures [21]. In both cases, the gold standard for PMMA-based intra-oral splints is the application of autopolymerizing PMMA in the powder–liquid technique.

With introduction of novel, light-curing resins [22], it remains questionable which way these medical devices can be manually additively securely fixed. Besides the conventional approach of adding MMA/PMMA in the powder–liquid technique, several manufactures offer “ready to use” or “fixation” resins for this objective. Furthermore, the conditioning of the concerning surfaces can be facilitated by chemical (i.e., MMA application) or physical (i.e., sandblasting) pretreatment [23,24].

However, the current literature lacks an insight into the adhesive bonding of such modelling resins to additive manufactured resin splints, depending on their pretreatment strategies and their status of clinical wear. This is crucial, because most of the splints are worn by night and the attached material must securely integrated, to hinder unintentional swallowing or even aspiration of fractured resin parts. Such an adverse event can be regarded as critical because the resins are not radiopaque and thereby cannot be localized or tracked in the patient’s body.

Therefore, an in-vitro study was set up to determine effects of surface conditioning as well simulation of hydrothermal ageing in the oral cavity on the shear bond strength of modelling resins to a 3D-printed splint material.

## 2. Materials and Methods

### 2.1. Specimen Preparation

**Figure 1:**
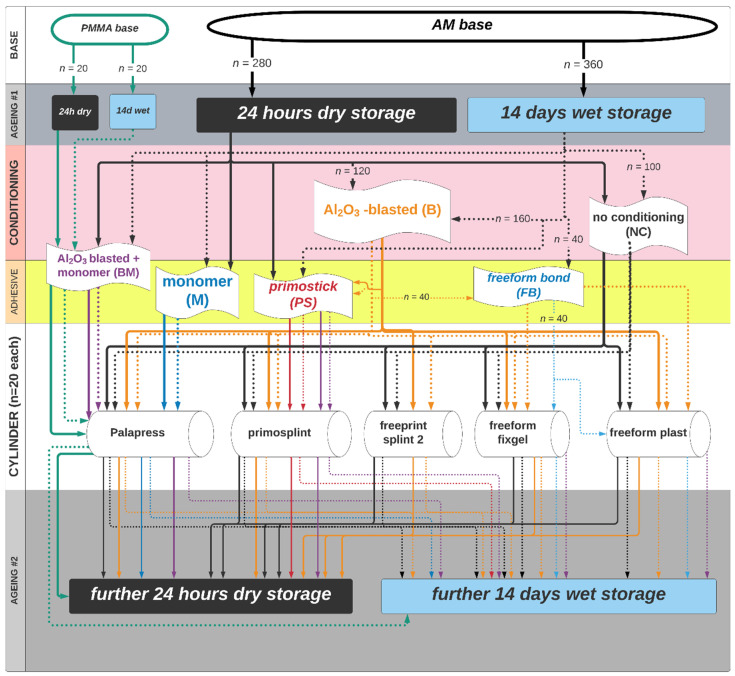
Flowchart of the specimen distribution over the experimental groups. PMMA-base and AM base were either aged by a wet storage in 37 °C distilled water for 14 days or not (24 h dry storage at 23 °C), followed by conditioning (B = Al_2_O_3_-blasted, M = acrylate monomer) or not (NC) and bonded to cylinders made from different polymers. There after every group was either stored for 14 more days in 37 °C distilled water or stored 24 h in dry condition before shear bond testing. A total of 680 specimens was distributed to 34 experimental groups (*n* = 20 each).

The study design is shown in the flowchart (Figure 1). There are two “base materials” (simulating the splint) and five “cylinders” (simulating the repair material). The conventional base group was made from polymethylmethacrylate (PMMA base) and the experimental base group a light curing 3D printable resin (AM base) for digital light processing (DLP). The base materials and cylinders were combined by adhesive bonding as shown in the flowchart with and without wet ageing of the base material. In total 680 specimens (*n* = 20 per group) were produced. All materials are further described in terms of their composition in Table S1 (Supplementary File 1: Tables S1–S5).

#### 2.1.1. Base Material and Ageing

For the AM base group, a total of 640 bar-shape specimens (20 × 10 × 2 mm^3^) were printed in with Rapidshape D 30 II (Rapid Shape Generative Production Systems GmbH, Heimsheim, Germany) from a splint material for digital light processing (Freeprint splint^®^ 2.0; DETAX GmbH& Co. KG, Ettlingen, Germany; LOT# 220405:04/21; see Supplementary Table S1). The dimensions of 20 × 10 × 2 mm^3^ of the specimen are demanded from the ISO standard 10477. The specimens were angulated 45° to the printing direction with a layer thickness of 50 µm (print-job available as digital object with doi:10.5281/zenodo.4926348). Postprocessing followed the respective instructions for use according to the materials’ manufacturer. At first, the so-called pre- and main-cleaning process was performed by storing the base materials in a tub filled with isopropanol 98%set in a running ultrasonic bath (Ultrasonic Cleaner; Proclean 10.0M ECO, ulsonix^®^ cleaning instruments, Zielona, Poland).

After 3 min of pre-cleaning, the specimens were shifted to a tub with unused isopropanol 98% for another 3 min ultrasonic cleaning. Subsequently, the base materials were dried with oil-free compressed air and post-exposed in a light curing unit (Otoflash G171, NK- OPTIK, Baierbrunn, Germany) with two times 2000 xenon light flashes under a nitrogen protected gas atmosphere. After the first 2000 flashes, the printed test specimen bases were turned over once to ensure light curing from both sides.

For the PMMA base group, 40 specimens were made from polymethylmethacrylate (PMMA) in powder (10 g) to liquid (7 mL) technique (Palapress clear, LOT monomer K010108:05/22, powder K010048:11/21; Heraeus Kulzer GmbH, Hanau, Germany) applying a preform of same dimensions made from a vinyl-polysiloxane mold polymerized submerged in water within a pressure pot at 2 bar for 20 min and 55 °C. Ten test specimen with the dimensions required by ISO standard 10477 were first designed with the aid of CAD and additively manufactured (freeprint splint^®^ 2.0 with Rapidshape D 30 II). These base specimens were arranged in two rows on a plastic shell and fixed at the edge with wax in order to be covered with liquid, autocuring vinyl-polysiloxane (Dubisil^®^ 30, Dreve Dentamid GmbH, Unna, Germany; LOT# 901659:01/21, 002693:02/22). The molded part was removed from the plastic tray as well as the test specimens. Thus, this negative molding could be used for the fabrication of the gold standard PMMA bases for the control group.

The 680 bar like specimen acted as the “base” and half of the specimens in the PMMA base were assigned to either “dry storage” (24 h at 23 °C in an airproof light-protected bag (Whirl-Pak^®^ black, HT83.1, Carl Roth GmbH & Co. KG, Karlsruhe, Germany) or “wet storage” (14 days at 37 °C in purified water protected from light, stored in an incubator (B6030, Heraeus Instruments, Bad Grund, Germany).

In the AM base group *n* = 280 were assigned to “dry storage” and *n* = 360 to “wet storage”. Both groups (PMMA and AM) with and without initial ageing were conditioned as follows after ageing.

#### 2.1.2. Base Material Surface Conditioning

The PMMA base material (*n* = 40) was blasted with 125µm Al_2_O_3_ (Cobra, Renfert GmbH, Hilzingen, Germany) at 3 bar for 10 s. Thereafter the monomer was applied with a microbrush for 30 s and let air dry for 30 s (Palapress liquid, Heraeus Kulzer GmbH).

The AM base material (*n* = 640) was conditioned as follows, experimental as well as according to instructions of the specific manufacturers:

- **AM base + BM (*n* = 40):** The AM base material was blasted with 125µm Al_2_O_3_ (Cobra, Renfert GmbH, Hilzingen, Germany) at 3 bar for 10 s. Thereafter the monomer was applied with a microbrush for 30 s and left to air dry for 30 s (Palapress liquid, Heraeus Kulzer GmbH).
- **AM base + M (*n* = 40):** The AM base material was conditioned with monomer (Palapress liquid, Heraeus Kulzer GmbH) applied with a microbrush for 30 s and left to air dry for 30 s.
- **AM base + B (*n* = 320):** The AM base material was blasted with 125µm Al_2_O_3_ (Cobra, Renfert GmbH, Hilzingen, Germany) at 3 bar for 10 s. Eighty out of these 320 were further conditioned with:
  *+PS (n = 40):* Primostick (PS202; LOT 201202:03/23, Primotec Joachim Mosch e.K, Bad Homburg, Germany) was applied for 30 s with a microbrush and light cured for 2 min (Elipar TriLight, 3M ESPE AG, Seefeld, Germany).
  *+FB (n = 40, only “wet storage”):* freeform bond liquid (freeform, LOT 200710:07/19; Detax GmbH & Co KG, Ettlingen, Germany) was applied for 30 s with a microbrush and let air dry for 30 s.
- **AM base + PS (*n* = 40):** Primostick (PS202; LOT 201202:03/23, Primotec Joachim Mosch e.K, Bad Homburg, Germany) was applied for 30 s with a microbrush and light cured for 2 min (Elipar TriLight, 3M ESPE AG, Seefeld, Germany).
- **AM base + FB (*n* = 40 only “wet storage”):** freeform^®^ bond liquid (freeform^®^ bond, LOT 200710:07/19; Detax GmbH & Co KG, Ettlingen, Germany) was applied to untreated AM base specimens for 30 s with a microbrush and left to air dry for 30 s.
  FB is provided by the manufacturer for adaptations to splints after a period of clinical application, only.
- **AM base + NC (*n* = 200).** The AM base material was connected “as printed” to the cylinders without any further surface treatment or conditioning.

### 2.2. Surface Characterization of the Bonding Interface

Three specimens from each base material group (surface conditioned and NC) were topographically evaluated to determine Ra (arithmetic average of filtered roughness profile) values. Therefore, contact profilometry was performed (Perthometer S6, Mahr GmbH, Göttingen, Germany) with 121 profile lines on a square of 9 mm^2^ applying a Gaussian filter of 0.6 mm analyzed with the attached software (Mountains Map V7.3, Digital Surf, Besançon, France). To avoid bias of measurements at the specimens’ marginal area, profiles 1–20 and 101–121 were excluded from statistical analysis.

For qualitative insights, one specimen per group was evaluated by SEM (LEO 1430 (Carl Zeiss AG, Oberkochen, Germany) after gold-palladium (SCD005, Bal-Tec GmbH, Schalksmühle, Germany) sputtering in 100×, 1000×, 2500×, and 5000× magnification.

### 2.3. Bonding of the Cylinder Specimens to the Base Materials

**Figure 2:**
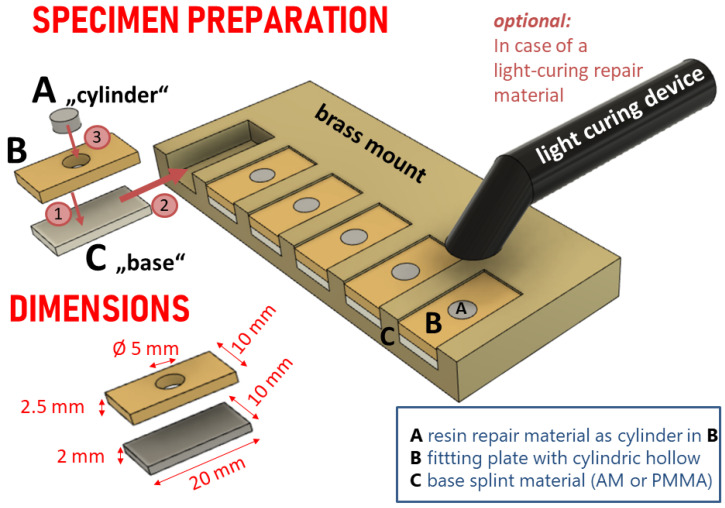
Schematic drawing of the device to facilitate the bonding of the repair material (**A**) within a cylinder to the base polymer (**C**) with help of a fitting plate (**B**). Fitting plate was laid on top of the base material and assembled in the slots of the brass mount. Thereafter the repair material was applied to the cylindric hollow in B. In case of light curing resin materials, it was performed initially with a handheld lamp on each specimen and the brass mount was put into the light oven afterwards. For PMMA, the brass mount was put into the pressure pot.

The base materials were set into the six slots of a brass mounting device (Figure 2). Each slot was covered with a fitting plate of 2.5 mm in height containing a cylindric hollow pattern (cylinder) of 5 mm in diameter in its centre. Mounts and plates were made from brass. Each specimen was noted with its base group and cylinder material on its rear side after bonding.

The PMMA base specimens were only bonded to auto curing PMMA mixing 10 g of powder to 7 mL of liquid (Palapress clear, LOT monomer K010108:05/22, powder K010048:11/21; Heraeus Kulzer GmbH) and polymerized submerged in water within a pressure pot at 2 bar for 20 min and 55 °C. This group of 40 specimens will be noted as the “gold standard” since it is the conventional method used in daily practice up to now.

The **AM base** materials were assigned as shown in the flowchart (Figure 1) to be bonded to cylinders of:

- **palapress** mixing 10 g of powder to 7 mL of liquid (Palapress clear, LOT monomer K010108:05/22, powder K010048:11/21; Heraeus Kulzer GmbH) and polymerized submerged in water within a pressure pot at 2 bar for 20 min and 55 °C.
- **primosplint** directly applied from the rods (Material primosplint; primotec^®^ Joachim Mosch e.K, Bad Homburg, Germany; LOT# 193138:08/21) to the cylinder with help of a Heidemann specular. Thereafter the material was light-cured for 1 min with direct application within the mold (Elipar TriLight, 3M ESPE AG) and 10 min without molding in a light oven (Speed Labolight, Hager & Werken GmbH & Co KG, Duisburg, Germany).
- **freeprint splint** was drawn up into a light-protected syringe (5 mL, BD Luer-Lok Tip REF 309649; Becton-Dickinson and Comp, Franklin Lakes, NJ, USA) from the bottle (Material Freeprint splint^®^ 2.0; DETAX GmbH & Co. KG, Ettlingen, Germany; LOT# 220405:04/21) and applied into the cylinder. Thereafter the material was light-cured for 1 min with direct light application within the mold (Elipar TriLight, 3M ESPE AG) and 10 min without molding in a light oven (Speed Labolight, Hager & Werken GmbH & Co KG, Duisburg, Germany).
- **freeform fixgel** was directly applied from the cartridge with the delivered mixing tip (Material Freeform fixgel^®^, DETAX GmbH& Co. KG, Ettlingen, Germany; LOT# 210801: 08/20) into the cylinder. Thereafter the material was light-cured for 1 min with direct light application within the mold (Elipar TriLight, 3M ESPE AG) and 10 min without molding in a light oven (Speed Labolight, Hager & Werken GmbH & Co KG, Duisburg, Germany).
- **freeform plast** was directly applied from the box (Material Freeform plast^®^, DETAX GmbH& Co. KG, Ettlingen, Germany; LOT# 210601: 06/20) to the cylinder with help of a Heidemann specular. Thereafter the material was light-cured for 1 min with a direct light application within the mold (Elipar TriLight, 3M ESPE AG) and 10 min without molding in a light oven (Speed Labolight, Hager & Werken GmbH & Co KG, Duisburg, Germany).

### 2.4. Artificial Ageing and Measurement of the Bonded Specimens

The specimens fabricated with dry storage base materials were assigned to further dry storage for 24 h as described above, and the base materials derived from “wet storage” were incubated for further 14 days, also as described above. Prior to shear bond testing, the bonding area of each specimen was determined by macroscopy-based measurements (M400, Wild, Herrburg, Switzerland) in 20× magnification applying the software Datinf Measure (Version 2.0; DatInf GmbH, Tübingen, Germany) in mm^2^ with three decimals as mean out of three repeated measurements.

### 2.5. Shear Bond Test and Fracture Mode Analysis

Fracture load (F, in Newton) was evaluated by a universal testing machine (Z010/TN2A, Zwick GmbH & Co. KG, Ulm, Germany) with the connected software (testXpert, Version 12, Zwick GmbH & Co. KG). The cross-head speed was set to 1 mm per minute from a starting distance of 0.5 mm to the cylinder, fixed in a 90° position to the piston (see Supplementary File 2, Figure S1). The shear bond strength (SBS, in MPa) was calculated as follows:

(1) $$\mathrm{SBS}\;=\frac{F}{A}$$

Equation (1) gives the calculation of shear bond strength (SBS) in MPa where *F* is the determined fracture load in Newton and *A* is the measured bonding area in mm^2^. In case of failure prior to the shear bond testing, the value is noted 0 and the specimen excluded from further statistical comparison of SBS.

The mode of fracture was determined visually with the macroscope (M400) in 20× magnification to decide if the fracture appeared adhesively (within the bonding surface), cohesively (within the specimen) or mixed (both present):

- Cohesive fracture: the fracture ran completely in the resin of the 3D printed base material.
- Adhesive fracture: the fracture ran between both materials (base material and repair material).
- Mixed fracture: the fracture contained both fracture modes. There were adhesively fractured areas and areas within the same specimen that were cohesively fractured.

### 2.6. Statistical Methods

The collected data were entered into a table and analyzed using the software package JMP, Version 154 (SAS Comp., Cary, NC, USA).

#### 2.6.1. Surface Roughness of Base Materials

Ra values were calculated as mean of 80 lines (lines #21-100 out of 121) per specimen to overcome the mismatch between circular bonding interface and the square of surface investigation. The mean and standard deviation will be given for each surface after dry and after wet storage.

The distributions are compared statistically as applying the non-parametric Wilcoxon test with one-way ChiSquare approximation was used (alpha = 0.05).

#### 2.6.2. Shear Bond Strength

The calculated shear bond values of each group (maximum *n* = 20 specimens) are described by mean, median, and standard deviation. The distribution of each group was tested for normality applying the Shapiro–Wilk test (alpha = 0.05). In case of normal distribution, the AM groups were compared to the PMMA-based “gold standard” with Dunnets test. Furthermore, a non-parametric comparison was performed with Wilcoxon for each pair rank sum test. Both tests were performed applying alpha = 0.05. A mean value of 5 MPa was set as the minimum threshold for clinical relevance, as given in the standard ISO 10477 [25].

#### 2.6.3. Failure Mode

The failure modes were described by relative frequencies for each group. Additionally, the mean SBS values within each group of specimens were calculated per failure mode.

## 3. Results

### 3.1. Surface Characteristics of the Base Bonding Interfaces

#### 3.1.1. Surface Roughness of Base Materials

The initial average surface roughness (Ra) of the bonding interfaces following conditioning is given in Table 1.

#### 3.1.2. Qualitative Surface Evaluation of Bonding Interface

Representative insights into the optical surface configuration in 1000× SEM magnification as well as 3D roughness profiles are given for the blasted and MMA-conditioned PMMA-base (Figure 3), the non-conditioned AM base (Figure 4), the sandblasted AM base (Figure 5), and the MMA-conditioned AM base (Figure 6). Further illustrations are provided in Figures S1–S7 within Supplementary File 2.

### 3.2. Shear Bond Strength

**Figure 7:**
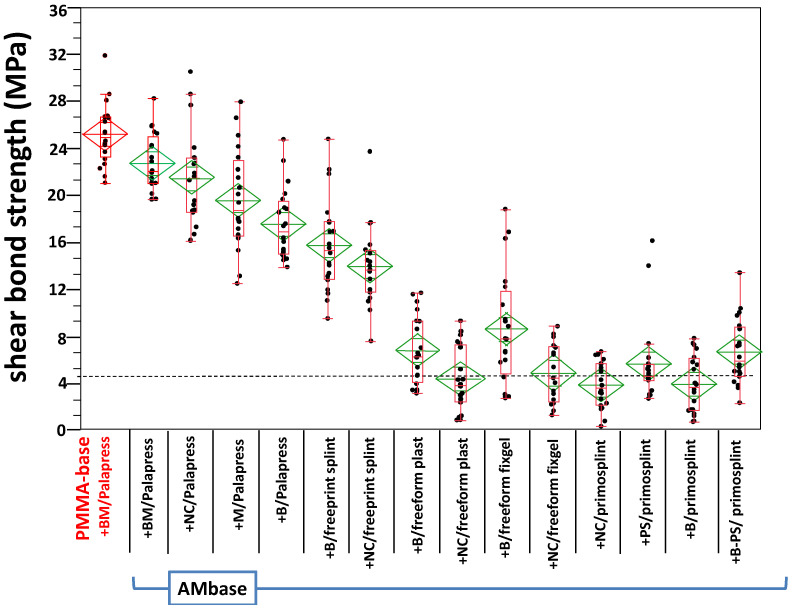
Box-plot of the shear bond strength values within the experimental groups after dry storage. The gold standard (PMMA) is plotted on the left (marked red). The boxplots with median values are supported by mean diamonds with 95% confidence intervals. The dashed horizontal line gives the threshold of 5 MPa defined by the standard (ISO 10477).

**Figure 8:**
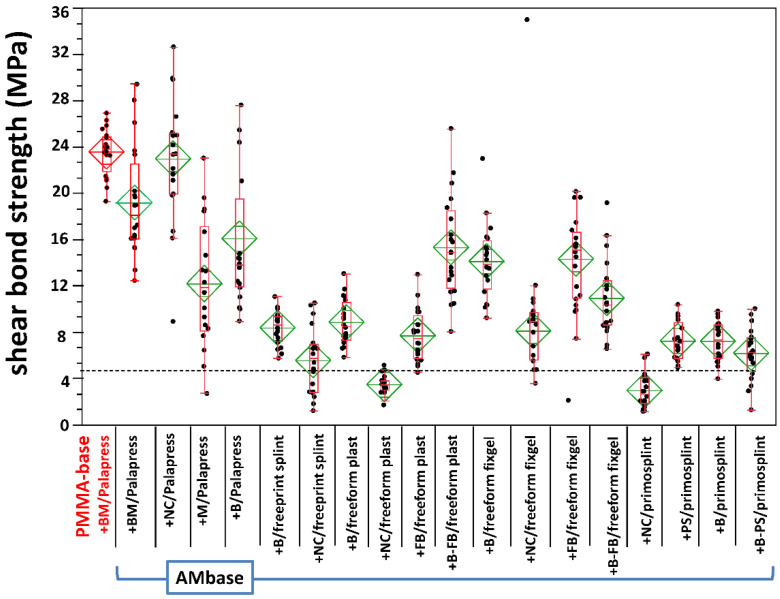
Boxplot of the shear bond strength values within the experimental groups after wet storage. The gold standard (PMMA) is plotted on the left (marked red). The boxplots with median values are supported by mean diamonds with 95% confidence intervals. The dashed horizontal line gives the threshold of 5 MPa defined by the standard (ISO 10477).

Six specimens failed prior to SBS testing; namely, two from AM base + M/palapress (wet storage), one from AM base + NC/palapress (wet storage), and three from the AM base + NC/freeform fixgel (dry storage). Thus, 674 specimens were successfully tested. The test for normality revealed a normal distribution for all groups; expect of AM base + PS/primosplint after dry storage. In this group, the test for normality revealed a non-normal distribution. The data is given in Appendix A and depicted in Figure 7 for dry storage and in Figure 8 for wet storage.

Dunnet’s test revealed no statistical significance between the gold standard (PMMA base + BM/Palapress) for AM base + BM/Palapress after dry storage and for AM base + NC/Palapress after wet storage (see Table S2, Supplementary File 2). The results of the multiple non-parametric comparison are given in Table S3 (dry storage) and Table S4 (wet storage) in Supplementary File 2. Four conditionings underwent with their mean value the threshold of 5 MPa.

### 3.3. Failure Modes

**Figure 9:**
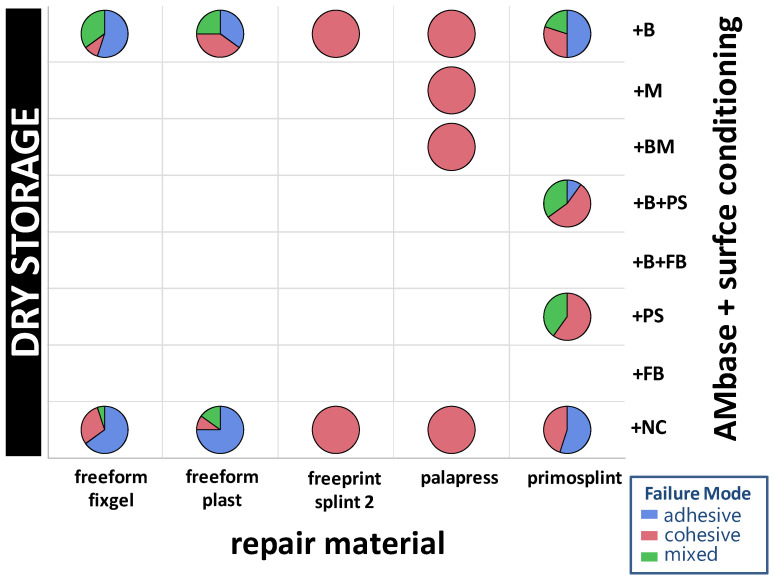
Distribution of failure modes of the repair materials (*x*-axis) against AM base splint material depending on the conditioning (right *y*-axis) after dry storage. Rectangles without pie charts were not performed in dry storage condition due to protocol.

**Figure 10:**
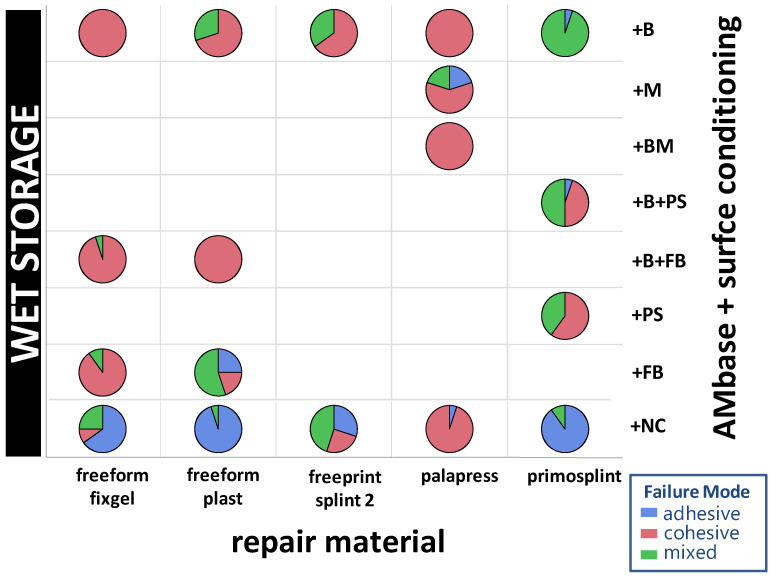
Distribution of failure modes of the repair materials (*x*-axis) against AM base splint material depending on the conditioning (right *y*-axis) after wet storage. Rectangles without pie charts were not performed in wet storage condition due to protocol.

In summary, 66 adhesive, 196 cohesive, and 35 mixed failure modes were observed in the 24 h dry group (23%, 65.3%, 11.7%) and in the 14 d wet group (17.9%, 59.7%, 22.4%), respectively. The distribution of failure modes within the experimental groups are given in Figure 9 and Figure 10.

The gold standard group (PMMA) has only cohesive fractures in the dry storage group (Mean SBS = 25.18 MPa) and in the wet storage group (Mean SBS = 23.57 MPa). Within die AM base group only cohesive fractures in both storage groups occurred in the specimen combined group AM base+BM/Palapress with *n* = 20 cohesive fractures for dry storage (Mean SBS = 22.7 MPa) and with *n* = 20 for the wet storage (Mean SBS = 19.18 MPa). Eventhough, only cohesive fractures were observed in the group AM-base+B/Palapress with *n* = 20 for dry storage (Mean SBS = 17.56) and wet storage (Mean SBS = 16.08 MPa) as well as in the s group AM base+NC/Palapress dry storage (*n* = 20; Mean SBS = 21.41 MPa) and wet storage (*n* = 19, Mean SBS = 22.97 MPa). In group AM base+B/primosplint wet storage revealed the highest mixed failure modes with *n* = 19 (Mean SBS = 7.39 MPa). The most adhesive fractures occurred in the group AM base+NC/freeform plast with dry storage *n*= 15 (Mean SBS = 3.75 MPa) and wet storage *n*= 19 (Mean SBS = 3.4 MPa). The SBS and failure mode data are given in Table S5 within Supplementary File 2.

## 4. Discussion

### 4.1. General Observations

The study revealed a gross variation of surface roughness in the AM base materials as well as within the shear bond strength of the repair materials bonded to the AM base. N-Thereby, the surface roughness Ra cannot be identified as a predictor for SBS against AM base, since low Ra values (as in non-conditioned AM base+NC) can reveal high SBS values (repair material = Palapress) and lowest SBS values (repair material = primosplint), too. This contradicts the rational of surface area enlargement by sandblasting [26], which is reported to enhance adhesive bonding of resins [27]. The additional presence of MMA enhances the effect, as also reported by Vallittu et al. [28]. Even if dry and wet storage surface characteristics revealed statistically significant differences, it has to be pointed out that a relevant difference could only be observed in AM base+PS.

Thus, chemical bonding seems to outweigh the physical properties (such as mechanical treatment with sandblasting) of the bonding interface. All the more, because in dry condition aluminum oxide blasting seems to enhance SBS values, except for Palapress.

Interestingly, the value of the gold standard PMMA sandblasted, and MMA conditioned repaired with PMMA was only reached by AM base in combination with Palapress (PMMA) as a repair material in non-conditioned or blasted and MMA conditioned surface. In addition to that, it is controversial that the tested bonding agents PS (primostick and FB (freeform bond) show inconsistent performance, not outperforming the MMA application.

These observations support the findings of Perea-Lowery and Vallittu [29], who investigated the bonding properties of conventional polyethylene and a 3D printed splint material to PMMA and applied a comparable ageing to the specimens. There were significant differences according to ageing but not for surface treatment. For 3D printed splint material the MMA group reached higher SBS values than the NC group, both in the range of this present study [29].

Comparing the SBS values of the present study with other investigations according to ISO 10477 most comparable is Ping Li et al. [30]. They investigated the effect of surface treatment (sandblasting and MMA) and artificial aging (thermal cycling in distilled water) on the SBS values of a 3D printed denture base resin (Freeprint) bonded to its polymerized base. In the non aged group, they found mean SBS values from 13.56 to 17.32 MPa whereas the aged group ranged between 6.67 and 16.73 MPa [30].

This range of shear bond strength was also reported by Younis et al. for veneering composite bonded to polyetheretherketone (PEEK) 5.38 MPa–10.04 MPa [31].

### 4.2. Specifics of Light Curing Material

The observation revealed that light curing repair materials were found to underperform with <10 MPa and even <5 MPa in mean SBS values. This was also found by Alkurt et al. [32], reporting a difference of about 50% in SBS values compared to autopolymerizing resins. As for freeform^®^ fixgel/- plast and primosplint the IFU indicates the application of a bonding agent (freeform^®^ bond for freeform^®^ fixgel and plast, primostick^®^ for primosplint) which contains MMA (see Table S1 in Supplementary File 1).

This was found to be superior to a non-conditioned (+NC) bonding interface repaired with primosplint (in dry and wet storage). The AM base group repaired with primosplint showed that the surface treatment by blasting and application of the bonding agent (with MMA) lead to higher SBS values then “blasted only” in the dry storage group. Within this group (AM base+B+PS/primosplint; dry storage) the number of cohesive failure modes increased compared to non-conditioned (+NC) and blasted (+B) bonding interface. This effect can be also detected in the AM base group repaired with freeform^®^ plast (wet storage). In this group the combination of blasting and bonding agent leads to higher SBS values and more cohesive fractures than in the non-treatment (+NC), blasted (+B), and bonding agent only (+FB) group. This effect was also described by Qaw et al., whereas specimens blasted with aluminumoxide and the application of bonding agents lead to higher SBS values between repair resin and denture base material [33].

With regard to the before mentioned and underlined by Curtis et al., reporting superior SBS values when using a bonding agent [34], it can be stated again, that the chemical bonding outweighs the physical surface treatment.

However, the inferior SBS values led to a further investigation of light transmission through repair materials filled into the cylinder for bonding. Therefore, a radiometer (Bluephase meter II, Ivoclar Vivadent AG, Schaan, Liechtenstein) detected the light intensity absorbed at the bonding interface and emitted by the handheld curing device (Elipar Tri Light, 3M Espe) for initial bonding (see Table 2) in these groups. Even with regard of the light curing post-processing in the light oven, this initial light intensity might have an impact on both, the bonding and mechanical properties, as reported for bulk fill resin composites [35].

As shown in Table 2, the brass cylinder restrains about 77% of light. This has to be further stressed by the fact that the initial power of this halogen-based polymerization device emits about 50% of the averaged power of current hand held devices, emitting up to 3000 mW/cm^2^ [36].

Therewith, the impact of light quantity on the shear bond strength of these repair materials should be further investigated. This aspect is crucial for medical device safety, especially when it comes to chair-side adaptations which call for a hand-held clinical device but offers no light oven.

### 4.3. Methodical Aspects and Limitations of the Study Design

Aside from the variety of materials and surface conditions the study has some methodical limitations as well as restrictions in clinical comparability.

The present study was conducted in accordance with the DIN EN ISO 10477 standard for polymer-based crown and bridge materials in dentistry. At the time of this study and publication, there was no corresponding ISO standard regarding the bond strength of repair materials/resins to additively manufactured splints. There are no defined test methods for testing the bond strength of polymers to conventionally manufactured splints in the DIN EN ISO standard 2795-2:2013 for “orthodontic resins and copolymers”.

Moreover, clinical aspects should also be taken into account when selecting and performing the test procedures. Occlusal splints are mainly used for therapy of craniomandibular disorders (TMD) and are exposed to shear and compression forces as well as two-body wear. Clinically, forces that most often lead to failure of the adhesive bonding between two materials are shear forces [22]. According to Sarac et al. and Qaw et al., measuring the bonding the shear test is regarded as an acceptable method, because it measures the force directly on the bonding interface as masticatory forces exert a force against this bonding site [24,33].

First, it must be mentioned that the dry storage group should simulate the dental lab situation of a fabricated splint being additionally modified by acrylic resin, prior to patient delivery. This storage time impairs the transferability of the findings to an immediate in-lab intervention (e.g., within 1–2 h) after post-processing of the splint. This aspect is not relevant for the wet storage group, which should simulate the situation of an already worn splint that may require corrections. However, herewith it remains unclear how the performance might be after 3 months or longer clinical performance. With respect to the ageing, it must be pointed out that the dry stored specimen was not further aged in wet conditions while the wet stored were further aged in wet conditions. This limits the insight into how the shear bond strength is changing when directly adapted splints are worn by the patient, as reported by Takahashi et al. [37]. This must be further investigated since UV polymerized materials seem to be more susceptible to water absorption than PMMA [38].

Moreover, the ageing did not cover temperature changes. This can lead to higher SBS values, but nocturnal bite guards are more likely to age in a wet environment of body temperature without variations of the environmental temperature [39]. This wet environment was simulated by distilled water isothermally at 37 °C in this study, and not by an artificial saliva, which was also reported to be equivalent Schulte et al. The authors postulate that artificial aging does not correspond to clinical aging, but no statistically significant differences between salvia and deionized water are to be expected [40].

Additionally, the chemical wear in relation to salvia is depending on various factors such as chemical composition, that with the use of salvia is the chemical wear is difficult to predict [41].

Within the AM base+FB (*n* = 40 only “wet storage”) the bonding agent freeform^®^ bond was only applied and investigated in the wet storage group in combination with the repair materials freeform^®^ fixgel and freeform^®^ plast because due to the manufacturer’s recommendations (DETAX GmbH & Co. KG) and instructions for use, the application of freeform^®^ bond is only essential on already completed and older samples, that were already worn. Therefore, the bonding agent freeform bond was only investigated in the wet storage group in which the repair material was freeform^®^ fixgel and freeform^®^ plast. Thus, materials are coordinated with each other and included in the freeform^®^ kit.

Preliminary tests revealed a difficulty to remove the fitting plate with the cylindric hollow (as shown in Figure 2B) when PMMA was used as a repair material. Thus, in these groups the rim of the cylindric hollow was insulate with a thin film of petrolatum (Vaseline Weiss, Bombastus- Werke AG, Freital, Germany; LOT# 10511009-6-0:01/21) using a microbrush. With regard to contamination of the bonding surface by residues of petrolatum jelly, the fitting plate brass fitting plate was cleaned on both sides with an alcohol-soaked paper towel before being placed on the base material. However, this necessity may have led to the three failing specimens prior to testing in the AM base+M/palapress (wet storage) group and theAM base+NC/palapress (wet storage) group. Thus, contamination of the bonding surface must be pointed out as a crucial aspect in daily practice.

Also, the clinical comparability is limited by the amount of repair material used (49 mm^3^), which is not the typical amount adapted to a surface of 20 mm^2^. This is especially crucial for the light curing resins discussed above. Danesh et al. described that with material layer depths of primosplint^®^ more than 9 mm not every tested specimen (cylinders with a diameter of 6 mm) was cured [42].

With regard to the gold standard, it can be critiqued that current PMMA-based splints are fabricated from industrial polymerized blanks and not by powder–liquid technique retaining a higher amount of MMA monomer [43,44,45]. In both cases (PMMA blanks and autopolymerizing PMMA), the surface treatment with sandblasting and MMA application can be regarded as the best in practice to ensure bonding of further autopolymerizing PMMA [46].

The selection of only one resin and one technology for additive manufacturing limits the possibility to convey data to other resins and 3D printing by SLA. The DLP fabrication is superior to SLA when it comes to time investment [47] and dimensional accuracy [48].

The chemical compositions of the 3D printing material depend on the AM technology and even when the same AM technology is used, the materials may differ in their post- processing techniques due to different manufacturers [49] or due to different devices [50].

Between SLA and DLP the main difference is the light source [51]. The DLP technology is similar to SLA technology and is classified in the same AM category by the American Section of the International Association for Testing Materials [52].

Nevertheless, a broader number of materials would have been tested with a comparable number of specimens, if the design of experiments would have been applied. In contrast to classical power calculation, such a statistical approach enables higher efficiency in output and is conductive to sustainability of biomaterial research.

This extends to the applied surface modifications of the bonding interface, e.g., by oxygen or nitrogen plasma. However, the surface modifications under evaluation are general available in dental labs and offices, worldwide. Therewith, the treatments are universally applicable, even in chair-side setting. Sophisticated approaches such as plasma or laser treatments may also carry an unrealistic efficiency due to disproportional cost-benefit ratio with regard to potential gains in adhesive bonding.

Surface roughness was investigated by contact profilometry as described in Section 2.2. The surface of the base material has been conditioned circularly at the position where the cylinders are attached to the base material. For this purpose, a fitting plate with a cylindric hollow was clamped onto the base of the specimen. The surface treatment was then carried out through the hollow of the fitting plate. Therewith, the needle of the profilometry testing machine measured partially outside the treated surface within a square of 3 × 3 mm^2^. In order to omit these measurements 20-line pairs on both sides were excluded from evaluation.

### 4.4. Outlook on Clinical Applicability

Digital dentistry and additive manufacturing allow not only the production of intraoral splints or dentures. There is also the possibility to produce digital wax-ups in order to preview the clinical outcome of restorations like veneers, crowns, and fixed dental prostheses in the anterior. Even if digital wax- up’s, which were milled or 3D printed, seem to outperform conventional produced wax-ups, there might be also the necessity of chair-side individualization by adding (light-curing) resin [53]. Moreover, a digitally fabricated splint can protect surgical areas that were treated with stem cells in guided bone regeneration within periimplantitis or jaw augmentation; and might need adjustment during healing process [54]. The secure addition (bonding) of resins to 3D printed appliances may also enable the later integration of sensors, in order to measure biting forces in occlusal splints or insertion torque of implants in drill guides [55,56].

## 5. Conclusions

The study revealed that the bonding interface of 3D printed resins has no impact on the shear bond strength of repair materials. 3D printed resins repaired with PMMA in powder–liquid mixing technique revealed an equivalent shear bond strength as bonded to conventional PMMA.

For the splint material fabricated in DLP, the sandblasting and application of MMA monomer is recommended after initial fabrication in the lab, and no further surface treatment is necessary when a splint was worn.

The use of light curing resin to repair or adapt an additively manufactures splint seems to carry a high risk of failure due to the low shear bond strength values. Sandblasting and adhesives are indicated, if worn splints will be modified by light curing resin. Even so, there might be an impact of the light amount for curing that needs further investigation.

## Figures and Tables

**Figure 3:**
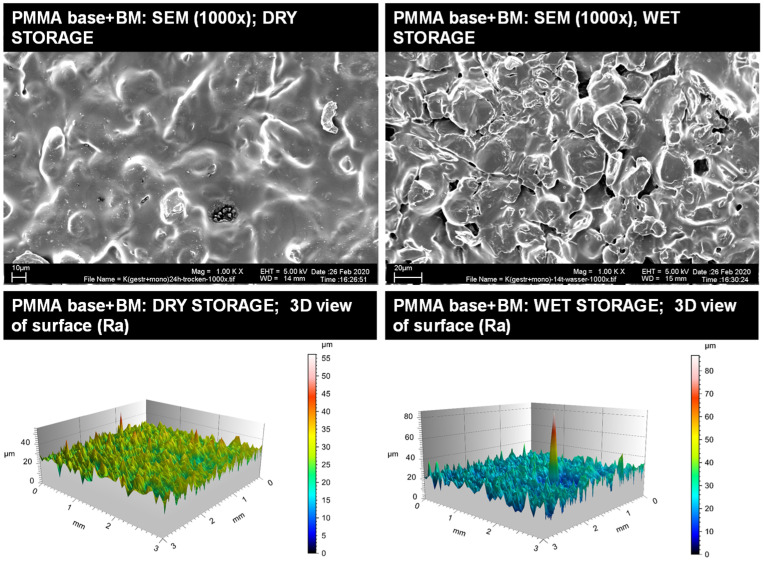
Qualitative insight of the gold standard (PMMA base, blasted and conditioned with MMA) via SEM imaging in 1000× magnification (**top**) and surface roughness profiles (**below**) for dry storage (**left**) and wet storage (**right**). Please note the differences in the scaling of the roughness profiles.

**Figure 4:**
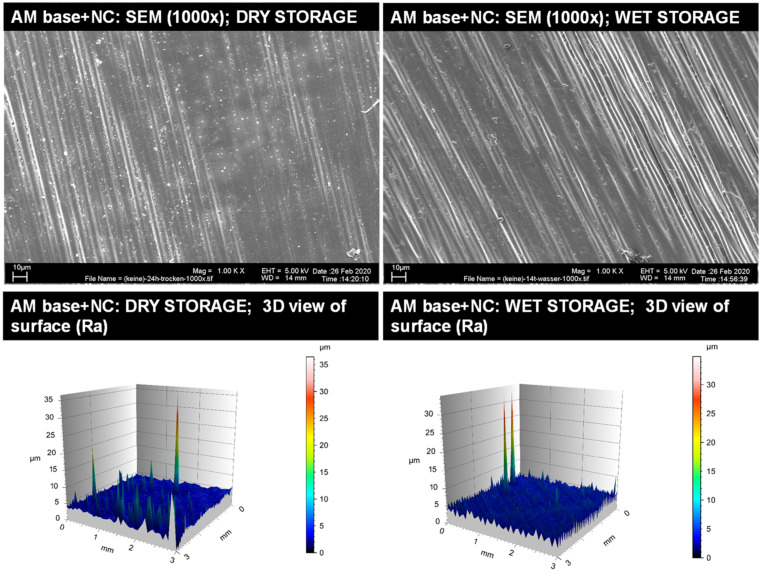
Qualitative insight of the AM base group with no surface condition (NC) via SEM imaging in 1000× magnification (**top**) and surface roughness profiles (**below**) for dry storage (**left**) and wet storage (**right**). Please note the differences in the scaling of the roughness profiles.

**Figure 5:**
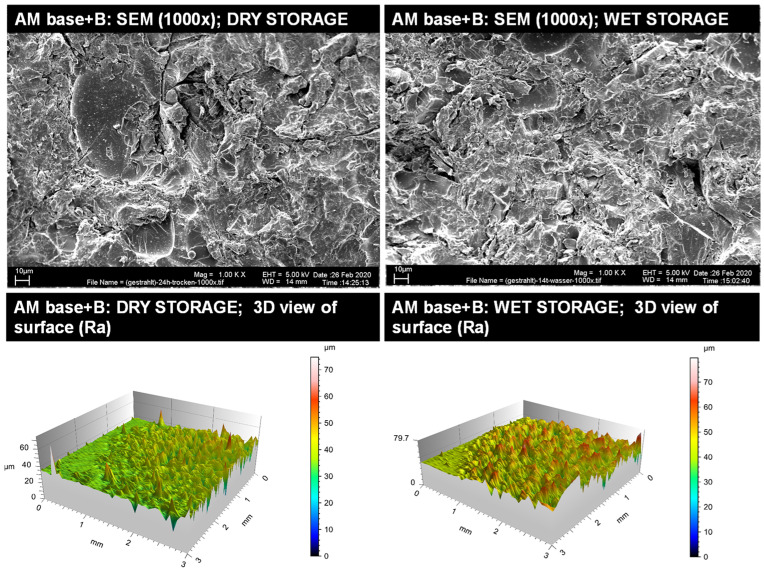
Qualitative insight of the AM base group blasted (B) via SEM imaging in 1000× magnification (**top**) and surface roughness profiles (**below**) for dry storage (**left**) and wet storage (**right**). Please note the differences in the scaling of the roughness profiles.

**Figure 6:**
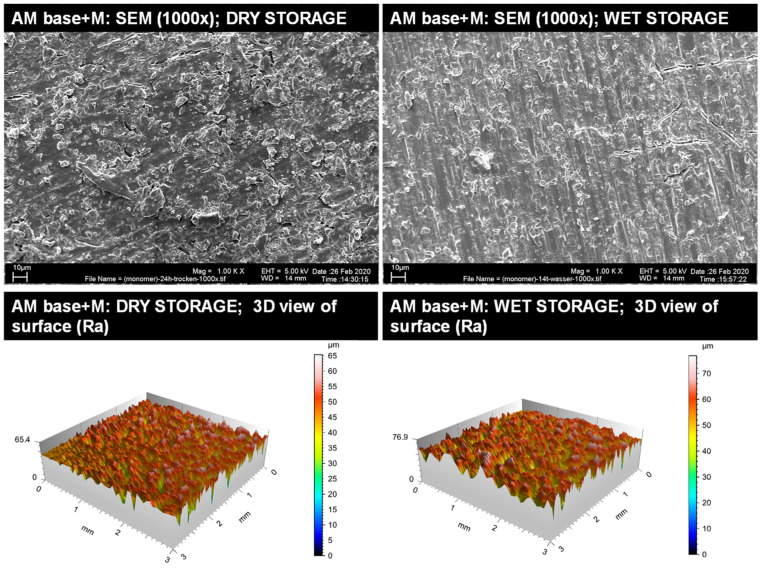
Qualitative insight of the AM base group conditioned with MMA (M) via SEM imaging in 1000× magnification (**top**) and surface roughness profiles (**below**) for dry storage (**left**) and wet storage (**right**). Please note the differences in the scaling of the roughness profiles.

**Table 1:** Surface roughness of the bonding interfaces at the base material in dry and wet condition. Statistical comparison by Wilcoxon Rank sum test and one-way chi-square approximation. (“n.a.” indicates groups that are not existent due to the study protocol).

Base Material and Conditioning	Dry Storage Ra (*n* Profile Lines ^1^, Mean, SD)	Wet Storage Ra (*n* Profile Lines ^1^, Mean, SD)	*p*-Value
PMMA base + BM	240, 3.37, 0.5	240, 3.69, 0.89	<0.0001
AM base + B	240, 4.89, 1.5	240, 6.23, 1.07	<0.0001
AM base + B-FB	0, n.a., n.a.	240, 0.77, 0.79	n.a.
AM base + BM	240, 5.23, 1.33	240, 5.89, 0.89	<0.0001
AM base + B-PS	240, 1.74, 1.08	240, 1.68, 2.17	<0.0001
AM base + FB	0, n.a., n.a.	240, 0.6, 0.7	n.a.
AM base + M	240, 0.93, 0.18	240, 0.5, 0.2	<0.0001
AM base + NC	240, 0.75, 0.39	240, 0.68, 0.31	<0.0001
AM base + PS	240, 1.13, 0.79	240, 0.26, 0.23	<0.0001

^1^ 80 profiles from three specimens in each group.

**Table 2:** Measurement of the light transmission through light-curing materials.

Light Curing Repair Material (Resin) 2.5 mm of Thickness	Light Intensity on the Bonding Interface mW/cm^2^ (Mean)
no material in cylinder (air)	780
freeform fixgel in brass cylinder without cylinder	125 560
freeform plast in brass cylinder without cylinder	113 510
Primosplint in brass cylinder without cylinder	116 480
freeprint splint in brass cylinder without cylinder	158 510

## Data Availability

Most of the data are provided with the supplementary files and in the cited repository. Further data are available upon reasonable request to the authors.

## Supplementary Materials

The following are available online at https://www.mdpi.com/article/10.3390/ma14143935/s1, Supplementary File 1: Table S1 Composition of materials, Table S2 Results of Dunnets test for statistical significance of shear bond strength (SBS) distributions, Table S3 Non-parametric multiple of the SBS distributions (dry storage), Table S4 Non-parametric multiple comparison of the SBS distributions (wet storage), Table S5 Failure mode and measured Shear Bond Strength; Supplementary File 2: Figure S1 Schematic drawing of the SBS testing; Figure S2–S9: SEM illustration and 3D profiles of the eight bonding interfaces; Printjob of specimens available with doi:10.5281/zenodo.4926348.

## Appendix A

**Table A1:** **SBS values of the tested specimens.** (n.a. indicated groups not tested due to the study protocol).

Base Material and Conditioning /Repair Material	Dry Storage SBS (*n*, Mean, SD)	Wet Storage SBS (*n*, Mean, SD)	SBS Difference Dry and Wet Storage (%)	Lower/Upper 95% –CI Dry Storage	Lower/Upper 95% –CI Wet Storage
PMMA base + BM/Palapress	20, 25.18, 2.59	20, 23.57, 2	−6.42	23.76/ 26.607	22.067/ 25.072
AM base + B/freeform plast	20, 6.87, 2.89	20, 8.85, 1.96	28.9	5.444/ 8.29	7.351/ 10.356
AM base + FB/freeform plast	n.a.	20, 7.71, 2.24	n.a.	n.a.	6.207/ 9.212
AM base + B-FB/freeform plast	n.a.	20, 15.32, 4.39	n.a.	n.a.	13.813/ 16.818
AM base+B/freeprint splint	20, 15.76, 3.94	20, 8.37, 1.43	−46.9	14.334/ 17.181	6.871/ 9.876
AM base+FB/freeform fixgel	n.a.	20, 14.31, 3.6	n.a.	n.a.	12.812/ 15.817
AM base+B/freeform fixgel	20, 8.65, 4.78	20, 14.12, 3.21	+63.3	7.222/ 10.068	12.615/ 15.62
AM base+B-FB/freeform fixgel	n.a.	20, 10.93, 3.26	n.a.	n.a.	9.425/ 12.43
AM base+PS/primosplint	20, 5.72, 3.4	20, 7.25,1.61	+26.7 *	4.3/ 7.147	5.747/ 8.752
AM base+B/primosplint	20, 4.01, 2.42	20, 7.22, 1.7	+80.2	2.583/ 5.43	5.715/ 8.72
AM base+B-PS/primosplint	20, 6.73, 2.79	20, 6.2, 2.23	−7.9 *	5.306/ 8.153	4.698/ 7.703
AM base+M/Palapress	20, 19.56, 4.19	18 **, 12.19, 5.51	−37.7	18.138/ 20.985	10.609/ 13.776
AM base+B/Palapress	20, 17.56, 3.02	20, 16.08, 6.91	−8.4 *	16.134/ 18.98	14.582/ 17.587
AM base+BM/Palapress	20, 22.7, 2.47	20, 19.18, 4.72	−15.5	21.275/ 24.122	17.674/ 20.679
AM base+NC/freeform fixgel	17 **, 4.93, 2.49	20, 8.11, 2.36	+64.3	3.39/ 6.477	6.607/ 9.612
AM base+NC/freeform plast	20, 4.45, 2.7	20, 3.48, 0.82	−21.8 *	3.028/ 5.875	1.981/ 4.986
AM base+NC/freeprint splint	20, 13.99, 3.31	20, 5.56, 2.83	−60.2	12.562/ 15.409	4.059/ 7.064
AM base+NC/Palapress	20, 21.41, 4.06	19 **, 22.97, 5.42	+7.3 *	19.989/ 22.836	21.43/ 24.513
AM base+NC/primosplint	20, 3.92, 1.97	20, 2.96, 1.46	−24.4 *	2.498/ 5.345	1.462/ 4.467

* indicates no statistical significant difference between dry and wet storage by students *t*-test. ** only the number of specimens were listed that were successfully tested with the shear bond strength.

## References

[1] Türp J.C., Komine F., Hugger A. (2004). Efficacy of stabilization splints for the management of patients with masticatory muscle pain: A qualitative systematic review. Clin. Oral Investig..

[2] Nishigawa K., Bando E., Nakano M. (2001). Quantitative study of bite force during sleep associated bruxism. J. Oral Rehabil..

[3] List T., Axelsson S. (2010). Management of TMD: Evidence from systematic reviews and meta-analyses. J. Oral Rehabil..

[4] Berntsen C., Kleven M., Heian M., Hjortsjo C. (2018). Clinical comparison of conventional and additive manufactured stabilization splints. Acta Biomater. Odontol. Scand..

[5] Bell R.B. (2018). A history of orthognathic surgery in north america. J. Oral Maxillofac. Surg..

[6] Rückschloß T., Ristow O., Müller M., Kühle R., Zingler S., Engel M., Hoffmann J., Freudlsperger C. (2019). Accuracy of patient-specific implants and additive-manufactured surgical splints in orthognathic surgery—A three-dimensional retrospective study. J. Craniomaxillofac. Surg..

[7] Wolowski A., Eger T., Braas R., Gohr J., Weber N., Witanski K., Wörner F. (2020). Long-term effects of splint therapy in patients with posttraumatic stress disease (PTSD). Clin. Oral Investig..

[8] Johansson A., Omar R., Carlsson G.E. (2011). Bruxism and prosthetic treatment: A critical review. J. Prosthodont. Res..

[9] Reyes-Sevilla M., Kuijs R.H., Werner A., Kleverlaan C.J., Lobbezoo F. (2018). Comparison of wear between occlusal splint materials and resin composite materials. J. Oral Rehabil..

[10] Baker P.S., Haywood V.B., Plummer K.D. (2007). Method for immediate fabrication of an occlusal device. J. Prosthet. Dent..

[11] Steele J.G., Wassell R.W., Walls A.W. (1992). A comparative study of the fit and retention of interocclusal splints constructed from heat-cured and autopolymerized polymethylmethacrylate. J. Prosthet. Dent..

[12] Bohnenkamp D.M. (1996). Dimensional stability of occlusal splints. J. Prosthet. Dent..

[13] Huettig F., Kustermann A., Kuscu E., Geis-Gerstorfer J., Spintzyk S. (2017). Polishability and wear resistance of splint material for oral appliances produced with conventional, subtractive, and additive manufacturing. J. Mech. Behav. Biomed. Mater..

[14] Marcel R., Reinhard H., Andreas K. (2020). Accuracy of CAD/CAM-fabricated bite splints: Milling vs. 3D printing. Clin. Oral Investig..

[15] Martorelli M., Gerbino S., Giudice M., Ausiello P. (2013). A comparison between customized clear and removable orthodontic appliances manufactured using RP and CNC techniques. Dent. Mater..

[16] Salmi M., Paloheimo K.S., Tuomi J., Ingman T., Mäkitie A. (2013). A digital process for additive manufacturing of occlusal splints: A clinical pilot study. J. R. Soc. Interface.

[17] Kessler A., Reymus M., Hickel R., Kunzelmann K.H. (2019). Three-body wear of 3D printed temporary materials. Dent. Mater..

[18] Kessler A., Reichl F.X., Folwaczny M., Högg C. (2020). Monomer release from surgical guide resins manufactured with different 3D printing devices. Dent. Mater..

[19] Quan H., Zhang T., Xu H., Luo S., Nie J., Zhu X. (2020). Photo-curing 3D printing technique and its challenges. Bioact. Mater..

[20] Fiedor P., Ortyl J. (2020). A new approach to micromachining: High-precision and innovative additive manufacturing solutions based on photopolymerization technology. Materials.

[21] Kerr W.J. (1984). Appliance breakages. Br. J. Orthod..

[22] Mariatos G., Frangou M., Polyzois G., Papadopoulos T. (2006). Evaluation of shear bond strength of microwaveable acrylic resins in denture repair: A comparative study. Acta Odontol. Scand..

[23] Mahadevan V., Krishnan M., Krishnan C.S., Azhagarasan N.S., Sampathkumar J., Ramasubramanian H. (2015). Influence of surface modifications of acrylic resin teeth on shear bond strength with denture base resin-an invitro study. J. Clin. Diagn. Res..

[24] Sarac Y.S., Sarac D., Kulunk T., Kulunk S. (2005). The effect of chemical surface treatments of different denture base resins on the shear bond strength of denture repair. J. Prosthet. Dent..

[25] (2018). Dentistry—Polymer-Based Crown and Veneering Materials.

[26] Akin H., Kirmali O., Tugut F., Coskun M.E. (2014). Effects of different surface treatments on the bond strength of acrylic denture teeth to polymethylmethacrylate denture base material. Photomed. Laser Surg..

[27] Jeong K.-W., Kim S.-H. (2019). Influence of surface treatments and repair materials on the shear bond strength of CAD/CAM provisional restorations. J. Adv. Prosthodont..

[28] Vallittu P.K., Lassila V.P., Lappalainen R. (1994). Wetting the repair surface with methyl methacrylate affects the transverse strength of repaired heat-polymerized resin. J. Prosthet. Dent..

[29] Perea-Lowery L., Vallittu P.K. (2019). Resin adjustment of three-dimensional printed thermoset occlusal splints: Bonding properties—Short communication. J. Mech. Behav. Biomed. Mater..

[30] Li P., Krämer-Fernandez P., Klink A., Xu Y., Spintzyk S. (2021). Repairability of a 3D printed denture base polymer: Effects of surface treatment and artificial aging on the shear bond strength. J. Mech. Behav. Biomed. Mater..

[31] Younis M., Unkovskiy A., ElAyouti A., Geis-Gerstorfer J., Spintzyk S. (2019). The Effect of various plasma gases on the shear bond strength between unfilled polyetheretherketone (PEEK) and veneering composite following artificial aging. Materials.

[32] Alkurt M., Yeşil Duymuş Z., Gundogdu M. (2014). Effect of repair resin type and surface treatment on the repair strength of heat-polymerized denture base resin. J. Prosthet. Dent..

[33] Qaw M.S., Abushowmi T.H., Almaskin D.F., AlZaher Z.A., Gad M.M., Al-Harbi F.A., Abualsaud R., Ammar M.M. (2020). A novel approach to improve repair bond strength of repaired acrylic resin: An in vitro study on the shear bond strength. J. Prosthodont..

[34] Curtis D.A., Eggleston T.L., Marshall S.J., Watanabe L.G. (1989). Shear bond strength of visible-light-cured resin relative to heat-cured resin. Dent. Mater..

[35] Besegato J.F., Jussiani E.I., Andrello A.C., Fernandes R.V., Salomão F.M., Vicentin B.L.S., Dezan-Garbelini C.C., Hoeppner M.G. (2019). Effect of light-curing protocols on the mechanical behavior of bulk-fill resin composites. J. Mech. Behav. Biomed. Mater..

[36] Shortall A.C., Hadis M.A., Palin W.M. (2021). On the inaccuracies of dental radiometers. PLoS ONE.

[37] Takahashi Y., Chai J., Kawaguchi M. (1998). Effect of water sorption on the resistance to plastic deformation of a denture base material relined with four different denture reline materials. Int. J. Prosthodont..

[38] Lin C.H., Lin Y.M., Lai Y.L., Lee S.Y. (2020). Mechanical properties, accuracy, and cytotoxicity of UV-polymerized 3D printing resins composed of Bis-EMA, UDMA, and TEGDMA. J. Prosthet. Dent..

[39] Lutz A.M., Hampe R., Roos M., Lümkemann N., Eichberger M., Stawarczyk B. (2019). Fracture resistance and 2-body wear of 3-dimensional-printed occlusal devices. J. Prosthet. Dent..

[40] Schulte J.K., Anderson G.C., Sakaguchi R.L., DeLong R. (1987). Wear resistance of isosit and polymethyl methacrylate occlusal splint material. Dent. Mater..

[41] Casey J., Dunn W.J., Wright E. (2003). In vitro wear of various orthotic device materials. J. Prosthet. Dent..

[42] Danesh G., Lippold C., Ziebura T., Reinhardt K.J., Schäfer E., Ehmer U. (2006). In-vitro investigation on suitability of light-cured resins for interocclusal splints: Part II: Surface hardness. J. Orofac. Orthop..

[43] Wiegand A., Stucki L., Hoffmann R., Attin T., Stawarczyk B. (2015). Repairability of CAD/CAM high-density PMMA- and composite-based polymers. Clin. Oral Investig..

[44] Rosca B., Ramalho S., Sampaio-Fernandes J.C., Portugal J. (2016). Reparability of two different CAD/CAM polymer materials using a light-cured composite and universal adhesives. Rev. Port. Estomatol. Med. Dentária Cir. Maxilofac..

[45] Edelhoff D., Schweiger J., Prandtner O., Trimpl J., Stimmelmayr M., Güth J.F. (2017). CAD/CAM splints for the functional and esthetic evaluation of newly defined occlusal dimensions. Quintessence Int..

[46] Lagouvardos P.E., Polyzois G.L. (2003). Shear bond strength between composite resin and denture teeth: Effect of tooth type and surface treatments. Int. J. Prosthodont..

[47] Zhang Z.C., Li P.L., Chu F.T., Shen G. (2019). Influence of the three-dimensional printing technique and printing layer thickness on model accuracy. J. Orofac. Orthop..

[48] Unkovskiy A., Bui P.H., Schille C., Geis-Gerstorfer J., Huettig F., Spintzyk S. (2018). Objects build orientation, positioning, and curing influence dimensional accuracy and flexural properties of stereolithographically printed resin. Dent. Mater..

[49] Reymus M., Fabritius R., Keßler A., Hickel R., Edelhoff D., Stawarczyk B. (2020). Fracture load of 3D-printed fixed dental prostheses compared with milled and conventionally fabricated ones: The impact of resin material, build direction, post-curing, and artificial aging-an in vitro study. Clin. Oral Investig..

[50] Alifui-Segbaya F., Bowman J., White A.R., George R., Fidan I., Love R.M. (2020). Chemical characterization of additively manufactured methacrylates for dental devices. Addit. Manuf..

[51] Revilla-León M., Meyers M.J., Zandinejad A., Özcan M. (2019). A review on chemical composition, mechanical properties, and manufacturing work flow of additively manufactured current polymers for interim dental restorations. J. Esthet. Restor. Dent..

[52] Revilla-León M., Özcan M. (2019). Additive manufacturing technologies used for processing polymers: Current status and potential application in prosthetic dentistry. J. Prosthodont..

[53] Cattoni F., Teté G., Calloni A.M., Manazza F., Gastaldi G., Capparè P. (2019). Milled versus moulded mock-ups based on the superimposition of 3D meshes from digital oral impressions: A comparative in vitro study in the aesthetic area. BMC Oral Health.

[54] Capparè P., Tetè G., Sberna M.T., Panina-Bordignon P. (2020). The emerging role of stem cells in regenerative dentistry. Curr. Gene Ther..

[55] Gao J., Liu L., Gao P., Zheng Y., Hou W., Wang J. (2019). Intelligent occlusion stabilization splint with stress-sensor system for bruxism diagnosis and treatment. Sensors.

[56] Capparé P., Vinci R., di Stefano D.A., Traini T., Pantaleo G., Gherlone E.F., Gastaldi G. (2015). Correlation between initial BIC and the insertion torque/depth integral recorded with an instantaneous torque-measuring implant motor: An in vivo study. Clin. Implant. Dent. Relat. Res..

