# Supplementary material for: Bonding Interface and Repairability of 3D-Printed Intraoral Splints: Shear Bond Strength to Current Polymers, with and without Ageing
Source: Materials (Basel). 2021 Jul 14;14(14):3935. doi: 10.3390/ma14143935 (PMC8307865; doi:10.3390/ma14143935)
Supplement: Supplementary file 1 [file materials-14-03935-s001.zip › Supplement_1_Table_S1-S5.pdf]

Table S1 Composition of Materials

Table S1 Composition of Materials

Product identifier:	Palapress® liquid		
Product code:	n. a.		
Lot number; Expiration date:	K010108; 16.05.2022		
Chemical Characterization:	Mixture based on methacrylates		
Components:	CAS No:	Quantity:	EG No:
methyl methacrylate (MMA)	80-62-6	>90%	201-297-1
tetramethylene dimethacrylate	2082-81-7	0- 5%	218-218-1
methyltrioctylammonium chloride	5137-55-3	<1%	225-896-2

Product identifier:	Palapress® powder		
Product code:	n. a.		
Lot number; Expiration date:	K010048; 07.11.2021		
Chemical Characterization:	Product based on methacrylate copolymers		
Components:	CAS No:	Quantity:	EG No:
Copolymerisat	25086-15-1	10- 25%	n. a.

Table S1 Composition of Materials

**Product identifier:**	primosplint, primotec®		
**Product code:**	1370110		
**Lot number; Expiration date:**	193138; 2021-08		
**Chemical Characterization:**	Mixture of acrylic resins, fillers and initiators		
**Light curing:**	-320 nm -400 nm<br>-UV- A- Light source		
**Components:**	**CAS No:**	**Quantity:**	**EG No:**
Urethane dimethacrylate (UDMA)	72869-86-4	20- 50%	n. a.
tricyclodecan dimethanol diacrylate (TCDDMDA)	42594-17-2	<20%	n. a.
Diphenyl (2,4,6-trimethylbenzoyl) phosphine oxide	75980-60-8	<1%	278-355-8

**Product identifier:**	freeform® fixgel		
**Product code:**	10340		
**Lot number; Expiration date:**	210801; 2020-08		
**Chemical Characterization:**	Mixture of acrylic/ methacrylic resins with auxilliary matters.		
**Light curing:**	-320 nm- 400 nm<br>-UV/ UV- A- Light source		
**Components:**	**CAS No:**	**Quantity:**	**EG No:**
tetrahydrofurfuryl methacrylate THFMA purified grade	2455-24-5	5- 25%	n. a.
Diphenyl (2,4,6-trimethylbenzoyl) phosphine oxide	75980-60-8	<1%	278-355-8

**Product identifier:**	freeform® plast		
**Product code:**	10257		
**Lot number; Expiration date:**	210601; 2020-06		
**Chemical Characterization:**	Mixture of acrylic/ methacrylic resins with auxilliary matters		
**Light curing:**	-320 nm- 400 nm<br>-UV/ UV- A- Light source		
**Components:**	**CAS No:**	**Quantity:**	**EG No:**
Tricyclodecane dimethanol diacrylate	42594-17-2	10- <15%	255-901-3
Diphenyl (2,4,6-trimethylbenzoyl) phosphine oxide	75980-60-8	<1%	278-355-8

Table S1 Composition of Materials

Product identifier:	primostick®		
Product code:	1370120		
Lot number; Expiration date:	201202; 2023-03		
Chemical Characterization:	-light curing bonding agent for primosplint<br>-Mixture of acrylic resins and initiators.		
Light curing:	-n. a.		
**Components:**	**CAS No:**	**Quantity:**	**EG No:**
methyl methacrylate;	80-62-6	20- 60%	201-297-1
acrylic resin	n. a.	<25%	n. a.
2-Propenoic acid, reaction products with pentaerythritol	1245638-61-2	<20%	629-850-6
Diphenyl (2,4,6-trimethylbenzoyl) phosphine oxide	75980-60-8	<3%	278-355-8

Product identifier:	freeform® bond		
Product code:	10571.2		
Lot number; Expiration date:	200710; 2019-07		
Chemical Characterization:	-bonding agent for ligth curing repair material<br>-Mixture of acrylic/ methacrylic resins with auxilliary matters		
Light curing:	-320 nm- 400 nm<br>-UV/ UV- A- Light source		
**Components:**	**CAS No:**	**Quantity:**	**EG No:**
methyl methacrylate, MMA; methyl 2-methylprop-2-enoate;	80-62-6	20- 70%	201-297-1
acrylic acid derivates	n. a.	25- 50%	n. a.
aliphatic polyestertriurethane triacrylate	n. a.	5- 20%	
vinylester resin	55818-57-0	1- <5%	n. a.
Diphenyl (2,4,6-trimethylbenzoyl) phosphine oxide	75980-60-8	0,1- 5%	278-355-8

Product identifier:	Freeprint ® splint 2.0 UV		
Product code:	1113		
Lot number; Expiration date:	220405; 2021-04		
Chemical Characterization:	light curing resin based on (meth) acrylate for additive manufacturing of splints		
Light curing:	-for DLP-printers with UV-LED 385 nm		
**Components:**	**CAS No:**	**Quantity:**	**EG No:**
isopropylidenediphenol peg-2 dimethacrylate	41637-38-1	90- <95%	n. a.
(5-ethyl-1,3-dioxan-5-yl) methyl acrylate	66492-51-1	1- <5%	n. a.
Diphenyl (2,4,6-trimethylbenzoyl) phosphine oxide	75980-60-8	1- <5%	015-203-00-X

Table S2 Results of Dunnets test for statistical significance of shear bond strength (SBS) distributions in the dry and wet storage group compared to the gold standard (PMMA-base+BM/Palapress) Positive values show pairs of means that are significantly different.

|d| = 2,83

DRY STORAGE

Comparison to PMMA-base+BM/Palapress	Abs(Dif)-LSD	p-Value
AM-base+BM/Palapress	-0,41	0,1363
AM-base+NC/Palapress	0,876	0,0033*
AM-base+M/Palapress	2,728	<,0001*
AM-base+B/Palapress	4,732	<,0001*
AM-base+B/freeprint splint 2	6,532	<,0001*
AM-base+NC/freeprint splint 2	8,303	<,0001*
AM-base+B/freeform fixgel	13,64	<,0001*
AM-base+B/freeform plast	15,42	<,0001*
AM-base+B-PS/primosplint	15,56	<,0001*
AM-base+PS/primosplint	16,57	<,0001*
AM-base+NC/freeform fixgel	17,23	<,0001*
AM-base+NC/freeform plast	17,84	<,0001*
AM-base+B/primosplint	18,28	<,0001*
AM-base+NC/primosplint	18,37	<,0001*

|d| = 2,89

WET STORAGE

Comparison to PMMA-base+BM/Palapress	Abs(Dif)-LSD	p-Value
AM-base+NC/Palapress	-2,57	1,0000
AM-base+BM/Palapress	1,266	0,0010*
AM-base+B/Palapress	4,358	<,0001*
AM-base+B-FB/freeform plast	5,127	<,0001*
AM-base+FB/freeform fixgel	6,128	<,0001*
AM-base+B/freeform fixgel	6,325	<,0001*
AM-base+M/Palapress	8,164	<,0001*
AM-base+B-FB/freeform fixgel	9,514	<,0001*
AM-base+B/freeform plast	11,59	<,0001*
AM-base+B/freeprint splint 2	12,07	<,0001*
AM-base+NC/freeform fixgel	12,33	<,0001*
AM-base+FB/freeform plast	12,73	<,0001*
AM-base+PS/primosplint	13,19	<,0001*
AM-base+B/primosplint	13,22	<,0001*
AM-base+B-PS/primosplint	14,24	<,0001*
AM-base+NC/freeprint splint 2	14,88	<,0001*
AM-base+NC/freeform plast	16,96	<,0001*
AM-base+NC/primosplint	17,48	<,0001*

Table S3 Non-parametric multiple comparison with Wilcoxon each pair test of the SBS distributions in the experimental groups with dry storage

Comparison of groups with dry storage (Table S3)		Score Mean Difference	Std Err Dif	Z	p-Value	Hodges-Lehmann	Lower CL	Upper CL
AM-base+M/Palapress	AM-base+B/freeform plast	19,9500	3,696846	5,39649	<,0001*	12,7736	10,0977	14,8004
AM-base+M/Palapress	AM-base+B/primosplint	19,9500	3,696846	5,39649	<,0001*	15,3607	13,0663	17,8206
AM-base+B/Palapress	AM-base+B/freeform plast	19,9500	3,696846	5,39649	<,0001*	10,6735	8,6239	12,5108
AM-base+B/Palapress	AM-base+B/primosplint	19,9500	3,696846	5,39649	<,0001*	13,3266	11,6129	15,0540
AM-base+B/Palapress	AM-base+B-PS/primosplint	19,9500	3,696846	5,39649	<,0001*	10,6184	9,0128	12,5204
AM-base+BM/Palapress	AM-base+B/freeform plast	19,9500	3,696846	5,39649	<,0001*	16,0275	14,1876	17,6860
AM-base+BM/Palapress	AM-base+B/freeform fixgel	19,9500	3,696846	5,39649	<,0001*	14,6530	11,9842	16,8673
AM-base+BM/Palapress	AM-base+PS/primosplint	19,9500	3,696846	5,39649	<,0001*	17,1120	15,8872	18,8508
AM-base+BM/Palapress	AM-base+B/primosplint	19,9500	3,696846	5,39649	<,0001*	18,6334	17,0790	20,1791
AM-base+BM/Palapress	AM-base+B-PS/primosplint	19,9500	3,696846	5,39649	<,0001*	15,9738	14,4404	17,7391
AM-base+NC/Palapress	AM-base+B/freeform plast	19,9500	3,696846	5,39649	<,0001*	14,0673	12,1836	16,7465
AM-base+NC/Palapress	AM-base+B/primosplint	19,9500	3,696846	5,39649	<,0001*	16,9304	15,0017	19,5288
AM-base+NC/Palapress	AM-base+B-PS/primosplint	19,9500	3,696846	5,39649	<,0001*	14,2506	12,2466	16,8935
AM-base+NC/Palapress	AM-base+NC/freeform plast	19,9500	3,696846	5,39649	<,0001*	16,3477	14,4777	19,1457
AM-base+NC/freeprint splint 2	AM-base+B/primosplint	19,8500	3,696846	5,36944	<,0001*	9,9253	7,9292	11,8088
AM-base+NC/Palapress	AM-base+PS/primosplint	19,8500	3,696846	5,36944	<,0001*	15,7489	13,6227	18,0012
AM-base+M/Palapress	AM-base+B-PS/primosplint	19,7500	3,696846	5,34239	<,0001*	12,6695	10,4615	15,1875
AM-base+NC/freeprint splint 2	AM-base+NC/freeform plast	19,6500	3,696846	5,31534	<,0001*	9,6671	7,3914	11,2099
AM-base+M/Palapress	AM-base+PS/primosplint	19,4500	3,696846	5,26124	<,0001*	13,5863	11,8634	16,3144
AM-base+B/freeprint splint 2	AM-base+B/freeform plast	19,2500	3,696846	5,20714	<,0001*	8,5865	6,4378	10,8550
AM-base+B/Palapress	AM-base+PS/primosplint	18,9500	3,696846	5,12599	<,0001*	11,8026	10,3378	13,8906
AM-base+NC/Palapress	AM-base+B/freeform fixgel	18,7500	3,696846	5,07189	<,0001*	13,1159	10,1049	15,7424
AM-base+NC/freeprint splint 2	AM-base+B-PS/primosplint	18,6500	3,696846	5,04484	<,0001*	7,2709	5,3790	8,9985
AM-base+NC/freeprint splint 2	AM-base+B/freeform plast	18,4500	3,696846	4,99074	<,0001*	7,2481	4,9406	8,9435
AM-base+NC/Palapress	AM-base+NC/freeform fixgel	18,4456	3,570783	5,16570	<,0001*	16,0105	14,0220	18,6941
AM-base+NC/freeprint splint 2	AM-base+NC/freeform fixgel	18,1191	3,570783	5,07427	<,0001*	8,8882	6,8989	10,9619
AM-base+M/Palapress	AM-base+B/freeform fixgel	17,9500	3,696846	4,85549	<,0001*	11,1347	8,4002	14,0475
AM-base+NC/Palapress	AM-base+NC/freeprint splint 2	17,5500	3,696846	4,74729	<,0001*	7,3806	5,0066	9,5176
AM-base+NC/freeprint splint 2	AM-base+PS/primosplint	17,1500	3,696846	4,63909	<,0001*	8,8337	7,1811	10,0853
AM-base+B/Palapress	AM-base+B/freeform fixgel	16,7500	3,696846	4,53089	<,0001*	9,3122	6,6823	11,8575
AM-base+BM/Palapress	AM-base+B/freeprint splint 2	16,4500	3,696846	4,44974	<,0001*	7,3101	5,1065	9,2449
AM-base+BM/Palapress	AM-base+B/Palapress	16,1500	3,696846	4,36859	<,0001*	5,3740	3,5535	6,8657
AM-base+NC/Palapress	AM-base+B/freeprint splint 2	14,1500	3,696846	3,82759	0,0001*	5,6141	3,1610	8,1512
AM-base+NC/freeprint splint 2	AM-base+B/freeform fixgel	12,7500	3,696846	3,44889	0,0006*	5,7789	3,1268	8,1932
AM-base+NC/Palapress	AM-base+B/Palapress	11,2500	3,696846	3,04314	0,0023*	3,6950	1,4621	6,2344
AM-base+B-PS/primosplint	AM-base+B/primosplint	10,1500	3,696846	2,74558	0,0060*	2,7359	0,9816	4,1840

Comparison of groups with dry storage (Table S3)		Score Mean Difference	Std Err Dif	Z	p-Value	Hodges-Lehmann	Lower CL	Upper CL
AM-base+M/Palapress	AM-base+B/freeprint splint 2	10,0500	3,696846	2,71853	0,0066*	3,7523	1,1116	6,5329
AM-base+BM/Palapress	AM-base+M/Palapress	9,5500	3,696846	2,58328	0,0098*	3,3043	0,9007	5,5475
AM-base+B/Palapress	AM-base+B/freeprint splint 2	6,4500	3,696846	1,74473	0,0810	1,9528	-0,3087	4,1817
AM-base+B-PS/primosplint	AM-base+PS/primosplint	6,2500	3,696846	1,69063	0,0909	1,1160	-0,2073	2,8608
AM-base+NC/Palapress	AM-base+M/Palapress	4,5500	3,696846	1,23078	0,2184	1,7916	-0,9408	4,6878
AM-base+NC/freeform fixgel	AM-base+B/primosplint	4,1897	3,570783	1,17333	0,2407	0,8823	-0,7705	2,6354
AM-base+B/freeform fixgel	AM-base+B/freeform plast	3,1500	3,696846	0,85208	0,3942	1,2554	-1,0339	4,0715
AM-base+NC/freeform plast	AM-base+B/primosplint	1,9500	3,696846	0,52748	0,5979	0,4394	-1,2681	2,0206
AM-base+B-PS/primosplint	AM-base+B/freeform plast	0,0500	3,696846	0,01353	0,9892	0,0070	-1,9823	1,6287
AM-base+NC/primosplint	AM-base+B/primosplint	0,0500	3,696846	0,01353	0,9892	0,0195	-1,5663	1,5026
AM-base+NC/freeform fixgel	AM-base+PS/primosplint	-1,7956	3,570783	-0,50286	0,6151	-0,4067	-2,0782	1,7713
AM-base+NC/freeform plast	AM-base+NC/freeform fixgel	-1,7956	3,570783	-0,50286	0,6151	-0,4156	-2,4770	1,2118
AM-base+NC/primosplint	AM-base+NC/freeform plast	-1,8500	3,696846	-0,50043	0,6168	-0,5111	-2,0237	1,3043
AM-base+B-PS/primosplint	AM-base+B/freeform fixgel	-4,1500	3,696846	-1,12258	0,2616	-1,5833	-4,0055	1,0622
AM-base+NC/primosplint	AM-base+NC/freeform fixgel	-4,4074	3,570783	-1,23428	0,2171	-0,9631	-2,6043	0,6518
AM-base+NC/freeform plast	AM-base+PS/primosplint	-5,2500	3,696846	-1,42013	0,1556	-1,1353	-2,4229	0,7073
AM-base+NC/freeprint splint 2	AM-base+B/freeprint splint 2	-5,2500	3,696846	-1,42013	0,1556	-1,5261	-3,9887	0,6495
AM-base+B/primosplint	AM-base+PS/primosplint	-5,3500	3,696846	-1,44718	0,1478	-1,3144	-2,8825	0,5727
AM-base+NC/Palapress	AM-base+BM/Palapress	-5,6500	3,696846	-1,52833	0,1264	-1,8301	-3,6412	0,8047
AM-base+NC/primosplint	AM-base+PS/primosplint	-5,6500	3,696846	-1,52833	0,1264	-1,1433	-2,4393	0,3699
AM-base+PS/primosplint	AM-base+B/freeform plast	-6,3500	3,696846	-1,71768	0,0859	-1,3469	-3,3729	0,3599
AM-base+B/Palapress	AM-base+M/Palapress	-6,3500	3,696846	-1,71768	0,0859	-1,8953	-4,4111	0,6057
AM-base+NC/freeform fixgel	AM-base+B/freeform plast	-6,6926	3,570783	-1,87428	0,0609	-2,0067	-3,8190	0,0670
AM-base+NC/freeform fixgel	AM-base+B-PS/primosplint	-6,8015	3,570783	-1,90476	0,0568	-1,7861	-3,3463	0,2140
AM-base+PS/primosplint	AM-base+B/freeform fixgel	-8,3500	3,696846	-2,25868	0,0239*	-2,6710	-4,8777	-0,2763
AM-base+NC/freeform fixgel	AM-base+B/freeform fixgel	-8,9779	3,570783	-2,51428	0,0119*	-3,2395	-5,8930	-0,5809
AM-base+NC/freeform plast	AM-base+B-PS/primosplint	-9,1500	3,696846	-2,47508	0,0133*	-2,2136	-4,1095	-0,6185
AM-base+NC/freeform plast	AM-base+B/freeform plast	-9,3500	3,696846	-2,52918	0,0114*	-2,3236	-4,1065	-0,4941
AM-base+B/primosplint	AM-base+B/freeform plast	-10,1500	3,696846	-2,74558	0,0060*	-2,7610	-4,6041	-1,0091
AM-base+NC/freeform plast	AM-base+B/freeform fixgel	-10,6500	3,696846	-2,88083	0,0040*	-3,6233	-6,3375	-1,4428
AM-base+BM/Palapress	PMMA-base+BM/Palapress	-10,9500	3,696846	-2,96198	0,0031*	-2,5307	-4,2424	-0,8453
AM-base+NC/primosplint	AM-base+B-PS/primosplint	-11,1500	3,696846	-3,01608	0,0026*	-2,6192	-4,1953	-1,0007
AM-base+NC/primosplint	AM-base+B/freeform plast	-11,4500	3,696846	-3,09724	0,0020*	-2,8271	-4,5543	-0,9617
AM-base+NC/Palapress	PMMA-base+BM/Palapress	-11,6500	3,696846	-3,15134	0,0016*	-4,0275	-6,1271	-1,8821
AM-base+B/primosplint	AM-base+B/freeform fixgel	-12,2500	3,696846	-3,31364	0,0009*	-4,1155	-6,6312	-1,6139
AM-base+NC/primosplint	AM-base+B/freeform fixgel	-13,0500	3,696846	-3,53004	0,0004*	-3,9556	-6,5442	-1,7522
AM-base+NC/freeprint splint 2	AM-base+B/Palapress	-13,3500	3,696846	-3,61119	0,0003*	-3,4901	-5,3084	-1,4927
AM-base+M/Palapress	PMMA-base+BM/Palapress	-14,6500	3,696846	-3,96284	<,0001*	-5,9248	-8,1674	-3,3501

Comparison of groups with dry storage (Table S3)		Score Mean Difference	Std Err Dif	Z	p-Value	Hodges-Lehmann	Lower CL	Upper CL
AM-base+NC/freeprint splint 2	AM-base+M/Palapress	-14,6500	3,696846	-3,96284	<,0001*	-5,4910	-8,0731	-2,9713
AM-base+B/freeform fixgel	AM-base+B/freeprint splint 2	-14,9500	3,696846	-4,04399	<,0001*	-7,3956	-9,9957	-4,7099
AM-base+PS/primosplint	AM-base+B/freeprint splint 2	-18,0500	3,696846	-4,88254	<,0001*	-10,0195	-12,2628	-8,1662
AM-base+NC/freeform fixgel	PMMA-base+BM/Palapress	-18,4456	3,570783	-5,16570	<,0001*	-20,0717	-22,0561	-18,3633
AM-base+NC/freeform fixgel	AM-base+B/freeprint splint 2	-18,4456	3,570783	-5,16570	<,0001*	-10,4371	-12,8887	-8,4291
AM-base+NC/freeform fixgel	AM-base+M/Palapress	-18,4456	3,570783	-5,16570	<,0001*	-14,4261	-16,9582	-11,9059
AM-base+NC/freeform fixgel	AM-base+B/Palapress	-18,4456	3,570783	-5,16570	<,0001*	-12,3449	-14,3124	-10,6394
AM-base+NC/freeform fixgel	AM-base+BM/Palapress	-18,4456	3,570783	-5,16570	<,0001*	-17,7764	-19,3947	-15,9787
AM-base+B/Palapress	PMMA-base+BM/Palapress	-18,4500	3,696846	-4,99074	<,0001*	-7,8008	-9,5209	-6,0447
AM-base+B/freeprint splint 2	PMMA-base+BM/Palapress	-18,5500	3,696846	-5,01779	<,0001*	-9,7101	-11,6603	-7,5434
AM-base+NC/freeprint splint 2	AM-base+BM/Palapress	-18,6500	3,696846	-5,04484	<,0001*	-8,6643	-10,5624	-7,0271
AM-base+B-PS/primosplint	AM-base+B/freeprint splint 2	-18,9500	3,696846	-5,12599	<,0001*	-8,6973	-11,2021	-6,7966
AM-base+NC/freeprint splint 2	PMMA-base+BM/Palapress	-19,3500	3,696846	-5,23419	<,0001*	-11,2690	-12,9680	-9,5420
AM-base+B/freeform plast	PMMA-base+BM/Palapress	-19,9500	3,696846	-5,39649	<,0001*	-18,2734	-20,2440	-16,3741
AM-base+B/freeform fixgel	PMMA-base+BM/Palapress	-19,9500	3,696846	-5,39649	<,0001*	-17,2290	-19,4488	-14,4897
AM-base+PS/primosplint	PMMA-base+BM/Palapress	-19,9500	3,696846	-5,39649	<,0001*	-19,9279	-21,4585	-18,4859
AM-base+B/primosplint	PMMA-base+BM/Palapress	-19,9500	3,696846	-5,39649	<,0001*	-21,0441	-22,8050	-19,3412
AM-base+B/primosplint	AM-base+B/freeprint splint 2	-19,9500	3,696846	-5,39649	<,0001*	-11,3623	-13,6935	-9,4392
AM-base+B-PS/primosplint	PMMA-base+BM/Palapress	-19,9500	3,696846	-5,39649	<,0001*	-18,6153	-20,3071	-16,7788
AM-base+NC/freeform plast	PMMA-base+BM/Palapress	-19,9500	3,696846	-5,39649	<,0001*	-20,9047	-22,6398	-18,9966
AM-base+NC/freeform plast	AM-base+B/freeprint splint 2	-19,9500	3,696846	-5,39649	<,0001*	-10,9709	-13,3216	-8,8462
AM-base+NC/freeform plast	AM-base+M/Palapress	-19,9500	3,696846	-5,39649	<,0001*	-14,9944	-17,2287	-12,6613
AM-base+NC/freeform plast	AM-base+B/Palapress	-19,9500	3,696846	-5,39649	<,0001*	-12,9758	-14,8520	-11,1884
AM-base+NC/freeform plast	AM-base+BM/Palapress	-19,9500	3,696846	-5,39649	<,0001*	-18,3003	-20,0534	-16,6285
AM-base+NC/primosplint	PMMA-base+BM/Palapress	-19,9500	3,696846	-5,39649	<,0001*	-21,0364	-22,6528	-19,6907
AM-base+NC/primosplint	AM-base+B/freeprint splint 2	-19,9500	3,696846	-5,39649	<,0001*	-11,2605	-13,5068	-9,4853
AM-base+NC/primosplint	AM-base+M/Palapress	-19,9500	3,696846	-5,39649	<,0001*	-15,2868	-17,8135	-13,2109
AM-base+NC/primosplint	AM-base+B/Palapress	-19,9500	3,696846	-5,39649	<,0001*	-13,1806	-15,0608	-11,7396
AM-base+NC/primosplint	AM-base+BM/Palapress	-19,9500	3,696846	-5,39649	<,0001*	-18,7020	-20,1093	-17,1273
AM-base+NC/primosplint	AM-base+NC/freeprint splint 2	-19,9500	3,696846	-5,39649	<,0001*	-9,7783	-11,4808	-8,2642
AM-base+NC/primosplint	AM-base+NC/Palapress	-19,9500	3,696846	-5,39649	<,0001*	-16,8684	-19,2838	-15,1748

Table S4 Non-parametric multiple comparison with Wilcoxon each pair test of the SBS distributions in the experimental groups with wet storage

Comparison of groups with wet storage (Table S4)		Score Mean Difference	Std Err Dif	Z	p-Value	Hodges-Lehmann	Lower CL	Upper CL
AM-base+BM/Palapress	AM-base+B/freeprint splint 2	19,9500	3,696846	5,39649	<,0001*	9,8369	7,8887	12,1553
AM-base+BM/Palapress	AM-base+PS/primosplint	19,9500	3,696846	5,39649	<,0001*	10,7204	9,2728	13,2993
AM-base+BM/Palapress	AM-base+B/primosplint	19,9500	3,696846	5,39649	<,0001*	10,9332	9,2196	13,5478
AM-base+BM/Palapress	AM-base+B-PS/primosplint	19,9500	3,696846	5,39649	<,0001*	12,0819	9,9707	14,5412
AM-base+BM/Palapress	AM-base+B/freeform plast	19,8500	3,696846	5,36944	<,0001*	9,3882	7,6943	12,0306
AM-base+BM/Palapress	AM-base+FB/freeform plast	19,8500	3,696846	5,36944	<,0001*	10,6962	8,8027	13,3010
AM-base+B/Palapress	AM-base+B/primosplint	19,6500	3,696846	5,31534	<,0001*	6,3670	4,9601	8,7276
AM-base+B/Palapress	AM-base+B-PS/primosplint	19,5500	3,696846	5,28829	<,0001*	7,6569	5,8068	10,3982
AM-base+NC/Palapress	AM-base+NC/freeform plast	19,4487	3,652685	5,32449	<,0001*	20,0296	17,8199	21,6695
AM-base+B/Palapress	AM-base+PS/primosplint	19,2500	3,696846	5,20714	<,0001*	6,6123	4,9018	8,5942
AM-base+NC/Palapress	AM-base+B/primosplint	19,1408	3,652685	5,24020	<,0001*	16,1580	13,8915	18,2965
AM-base+NC/Palapress	AM-base+B-PS/primosplint	19,1408	3,652685	5,24020	<,0001*	17,1218	14,7142	19,3014
AM-base+NC/Palapress	AM-base+NC/freeprint splint 2	19,1408	3,652685	5,24020	<,0001*	18,0175	14,9025	20,3822
AM-base+B/freeform fixgel	AM-base+B/freeprint splint 2	19,0500	3,696846	5,15304	<,0001*	5,6531	4,0930	7,0105
AM-base+NC/Palapress	AM-base+FB/freeform plast	18,9355	3,652685	5,18400	<,0001*	15,5076	13,3679	18,0461
AM-base+NC/Palapress	AM-base+PS/primosplint	18,9355	3,652685	5,18400	<,0001*	16,0143	14,0691	18,2468
AM-base+B/freeform fixgel	AM-base+FB/freeform plast	18,5500	3,696846	5,01779	<,0001*	6,3902	4,5785	8,1423
AM-base+B/Palapress	AM-base+B/freeprint splint 2	18,5500	3,696846	5,01779	<,0001*	5,3326	3,8539	7,2784
AM-base+NC/Palapress	AM-base+B/freeform plast	18,5250	3,652685	5,07161	<,0001*	14,4621	12,2078	16,7512
AM-base+NC/Palapress	AM-base+B/freeprint splint 2	18,4224	3,652685	5,04351	<,0001*	15,0582	12,7735	16,8896
AM-base+B/Palapress	AM-base+FB/freeform plast	18,3500	3,696846	4,96369	<,0001*	6,2576	4,4126	8,6586
AM-base+NC/Palapress	AM-base+NC/freeform fixgel	18,3197	3,652685	5,01542	<,0001*	15,2350	12,6371	17,4491
AM-base+B-FB/freeform plast	AM-base+FB/freeform plast	18,2500	3,696846	4,93664	<,0001*	7,1447	5,1031	9,6762
AM-base+NC/Palapress	AM-base+B-FB/freeform fixgel	17,8066	3,652685	4,87493	<,0001*	12,8557	9,5459	15,0506
AM-base+FB/freeform fixgel	AM-base+FB/freeform plast	17,6500	3,696846	4,77434	<,0001*	6,7338	4,5526	9,0237
AM-base+FB/freeform fixgel	AM-base+B/freeprint splint 2	17,6500	3,696846	4,77434	<,0001*	6,1768	4,0505	7,8755
AM-base+BM/Palapress	AM-base+B-FB/freeform fixgel	17,3500	3,696846	4,69319	<,0001*	7,7566	5,5534	10,2519
AM-base+B-FB/freeform plast	AM-base+B/freeform plast	17,2500	3,696846	4,66614	<,0001*	5,9296	3,9044	8,3708
AM-base+B/freeform fixgel	AM-base+B/freeform plast	17,2500	3,696846	4,66614	<,0001*	5,1604	3,4304	6,8904
AM-base+B/Palapress	AM-base+B/freeform plast	17,1500	3,696846	4,63909	<,0001*	5,1048	3,2197	7,1464
AM-base+NC/Palapress	AM-base+FB/freeform fixgel	16,1645	3,652685	4,42537	<,0001*	9,0820	5,8514	11,7996
AM-base+NC/Palapress	AM-base+B/freeform fixgel	16,1645	3,652685	4,42537	<,0001*	9,4769	6,6787	11,7289
AM-base+FB/freeform fixgel	AM-base+B/freeform plast	16,0500	3,696846	4,34154	<,0001*	5,6516	3,3400	7,8430
AM-base+NC/Palapress	AM-base+M/Palapress	15,6330	3,560327	4,39090	<,0001*	11,4154	6,8251	15,0101

Comparison of groups with wet storage (Table S4)		Score Mean Difference	Std Err Dif	Z	p-Value	Hodges-Lehmann	Lower CL	Upper CL
AM-base+NC/Palapress	AM-base+B-FB/freeform plast	14,3171	3,652685	3,91961	<,0001*	8,3835	4,8386	11,2620
AM-base+BM/Palapress	AM-base+B/freeform fixgel	13,5500	3,696846	3,66529	0,0002*	4,5589	2,0992	6,8951
AM-base+M/Palapress	AM-base+B-PS/primosplint	13,1417	3,610556	3,63979	0,0003*	5,6345	2,5059	8,9464
AM-base+BM/Palapress	AM-base+M/Palapress	12,6139	3,610556	3,49361	0,0005*	6,9363	3,2656	10,3574
AM-base+B-FB/freeform fixgel	AM-base+FB/freeform plast	12,4500	3,696846	3,36774	0,0008*	2,8715	1,2471	4,6626
AM-base+BM/Palapress	AM-base+FB/freeform fixgel	11,5500	3,696846	3,12429	0,0018*	4,3033	1,4849	7,5404
AM-base+B/Palapress	AM-base+B-FB/freeform fixgel	11,2500	3,696846	3,04314	0,0023*	3,5171	1,4453	5,8061
AM-base+M/Palapress	AM-base+PS/primosplint	11,2417	3,610556	3,11356	0,0018*	4,2830	1,7635	7,6873
AM-base+NC/Palapress	AM-base+B/Palapress	11,1355	3,652685	3,04859	0,0023*	8,9186	4,3681	11,5250
AM-base+M/Palapress	AM-base+B/primosplint	10,9250	3,610556	3,02585	0,0025*	4,4986	1,6387	7,9209
AM-base+M/Palapress	AM-base+FB/freeform plast	9,9750	3,610556	2,76273	0,0057*	3,8915	1,2561	7,4325
AM-base+B-FB/freeform fixgel	AM-base+B/freeprint splint 2	9,9500	3,696846	2,69148	0,0071*	1,9925	0,5721	3,6764
AM-base+BM/Palapress	AM-base+B/Palapress	9,8500	3,696846	2,66443	0,0077*	4,1740	1,5120	6,5656
AM-base+BM/Palapress	AM-base+B-FB/freeform plast	9,5500	3,696846	2,58328	0,0098*	3,7792	0,8392	6,5654
AM-base+NC/Palapress	AM-base+BM/Palapress	9,3908	3,652685	2,57093	0,0101*	4,6465	0,8696	7,7198
AM-base+NC/freeform fixgel	AM-base+B-PS/primosplint	8,4500	3,696846	2,28573	0,0223*	2,0815	0,3314	3,4627
AM-base+M/Palapress	AM-base+B/freeprint splint 2	8,2861	3,610556	2,29497	0,0217*	3,3133	0,3469	6,6272
AM-base+NC/freeprint splint 2	AM-base+NC/freeform plast	7,9500	3,696846	2,15048	0,0315*	2,0111	0,0962	3,3564
AM-base+B-FB/freeform fixgel	AM-base+B/freeform plast	7,7500	3,696846	2,09638	0,0360*	1,5970	0,1950	3,3082
AM-base+M/Palapress	AM-base+B/freeform plast	7,6528	3,610556	2,11956	0,0340*	2,7425	0,1197	6,1725
AM-base+B/Palapress	AM-base+M/Palapress	6,3861	3,610556	1,76873	0,0769	2,9512	-0,4643	6,7093
AM-base+NC/freeform fixgel	AM-base+B/primosplint	5,4500	3,696846	1,47423	0,1404	0,9615	-0,3123	2,5409
AM-base+B/freeprint splint 2	AM-base+FB/freeform plast	5,1500	3,696846	1,39308	0,1636	0,8651	-0,5104	2,0667
AM-base+NC/freeform fixgel	AM-base+PS/primosplint	4,7500	3,696846	1,28488	0,1988	1,1119	-0,4323	2,5002
AM-base+NC/freeform fixgel	AM-base+FB/freeform plast	2,3500	3,696846	0,63568	0,5250	0,6374	-0,9538	2,3262
AM-base+M/Palapress	AM-base+B-FB/freeform fixgel	2,1639	3,610556	0,59932	0,5490	1,0204	-2,0883	4,4217
AM-base+B/primosplint	AM-base+PS/primosplint	0,1500	3,696846	0,04058	0,9676	0,0206	-1,1177	1,1825
AM-base+B/Palapress	AM-base+B/freeform fixgel	0,0000	3,696846	0,00000	1,0000	0,0072	-1,8714	3,1551
AM-base+NC/freeform fixgel	AM-base+B/freeprint splint 2	0,0000	3,696846	0,00000	1,0000	0,0005592	-1,4880	1,2070
AM-base+B/Palapress	AM-base+FB/freeform fixgel	-0,3500	3,696846	-0,09468	0,9246	-0,1469	-2,6567	3,6546
AM-base+NC/Palapress	PMMA-base+BM/Palapress	-0,5645	3,652685	-0,15454	0,8772	-0,1983	-2,5511	1,7233
AM-base+B/Palapress	AM-base+B-FB/freeform plast	-1,5500	3,696846	-0,41928	0,6750	-0,5084	-3,3863	2,4409
AM-base+PS/primosplint	AM-base+FB/freeform plast	-1,6500	3,696846	-0,44633	0,6554	-0,3386	-1,5654	0,9497
AM-base+B/freeform fixgel	AM-base+FB/freeform fixgel	-1,8500	3,696846	-0,50043	0,6168	-0,5043	-2,6250	2,0145
AM-base+B/primosplint	AM-base+FB/freeform plast	-1,8500	3,696846	-0,50043	0,6168	-0,2466	-1,6746	0,9426
AM-base+FB/freeform fixgel	AM-base+B-FB/freeform plast	-2,0500	3,696846	-0,55453	0,5792	-0,6190	-3,4422	1,9753
AM-base+B/freeprint splint 2	AM-base+B/freeform plast	-2,4500	3,696846	-0,66273	0,5075	-0,3077	-1,6694	0,8412
AM-base+NC/freeform fixgel	AM-base+B/freeform plast	-2,5500	3,696846	-0,68978	0,4903	-0,6047	-2,1334	1,1386

Comparison of groups with wet storage (Table S4)		Score Mean Difference	Std Err Dif	Z	p-Value	Hodges-Lehmann	Lower CL	Upper CL
AM-base+B/freeform fixgel	AM-base+B-FB/freeform plast	-3,1500	3,696846	-0,85208	0,3942	-1,1085	-3,4328	1,4004
AM-base+NC/freeprint splint 2	AM-base+B-PS/primosplint	-3,4500	3,696846	-0,93323	0,3507	-0,7383	-2,6827	0,9543
AM-base+B-PS/primosplint	AM-base+PS/primosplint	-4,8500	3,696846	-1,31193	0,1895	-0,9520	-2,3007	0,4057
AM-base+B-PS/primosplint	AM-base+B/primosplint	-4,8500	3,696846	-1,31193	0,1895	-1,0026	-2,3589	0,4344
AM-base+M/Palapress	AM-base+B/freeform fixgel	-5,2250	3,610556	-1,44715	0,1479	-2,0846	-5,1948	1,2712
AM-base+M/Palapress	AM-base+FB/freeform fixgel	-5,3306	3,610556	-1,47638	0,1398	-2,2465	-6,0085	1,1816
AM-base+NC/primosplint	AM-base+NC/freeform plast	-5,5500	3,696846	-1,50128	0,1333	-0,7487	-1,4591	0,2425
AM-base+FB/freeform plast	AM-base+B/freeform plast	-6,1500	3,696846	-1,66358	0,0962	-1,2553	-2,5446	0,2621
AM-base+B-PS/primosplint	AM-base+FB/freeform plast	-6,2500	3,696846	-1,69063	0,0909	-1,2306	-2,8353	0,1450
AM-base+M/Palapress	AM-base+B-FB/freeform plast	-6,5972	3,610556	-1,82720	0,0677	-3,1422	-6,5416	0,3554
AM-base+NC/freeprint splint 2	AM-base+B/primosplint	-6,8500	3,696846	-1,85293	0,0639	-1,8851	-3,2954	0,0845
AM-base+PS/primosplint	AM-base+B/freeprint splint 2	-7,3500	3,696846	-1,98818	0,0468*	-1,1877	-2,2638	-0,0399
AM-base+B/primosplint	AM-base+B/freeprint splint 2	-7,7500	3,696846	-2,09638	0,0360*	-1,0685	-2,3222	-0,1095
AM-base+NC/freeprint splint 2	AM-base+PS/primosplint	-7,8500	3,696846	-2,12343	0,0337*	-2,0146	-3,4465	-0,1103
AM-base+NC/freeprint splint 2	AM-base+FB/freeform plast	-8,0500	3,696846	-2,17753	0,0294*	-2,2279	-3,8076	-0,2982
AM-base+B/primosplint	AM-base+B/freeform plast	-8,3500	3,696846	-2,25868	0,0239*	-1,5658	-2,7194	-0,3561
AM-base+NC/freeform fixgel	AM-base+M/Palapress	-8,3917	3,610556	-2,32420	0,0201*	-3,6617	-7,3608	-0,5333
AM-base+NC/freeform fixgel	AM-base+B-FB/freeform fixgel	-8,8500	3,696846	-2,39393	0,0167*	-2,3140	-4,3965	-0,5798
AM-base+NC/freeprint splint 2	AM-base+NC/freeform fixgel	-9,7500	3,696846	-2,63738	0,0084*	-2,7351	-4,3317	-0,7561
AM-base+PS/primosplint	AM-base+B/freeform plast	-9,8500	3,696846	-2,66443	0,0077*	-1,5948	-2,6856	-0,3052
AM-base+B-FB/freeform fixgel	AM-base+FB/freeform fixgel	-10,3500	3,696846	-2,79968	0,0051*	-3,6166	-6,0922	-1,1224
AM-base+B-FB/freeform fixgel	AM-base+B/freeform fixgel	-10,5500	3,696846	-2,85378	0,0043*	-3,4737	-5,2856	-1,3851
AM-base+NC/primosplint	AM-base+NC/freeprint splint 2	-11,1500	3,696846	-3,01608	0,0026*	-2,4897	-4,2852	-0,7636
AM-base+NC/freeprint splint 2	AM-base+B/freeprint splint 2	-11,4500	3,696846	-3,09724	0,0020*	-2,8921	-4,5151	-1,2905
AM-base+B-PS/primosplint	AM-base+B/freeprint splint 2	-11,6500	3,696846	-3,15134	0,0016*	-2,0966	-3,2653	-0,8713
AM-base+B-FB/freeform fixgel	AM-base+B-FB/freeform plast	-11,7500	3,696846	-3,17839	0,0015*	-4,2940	-6,7412	-1,8631
AM-base+BM/Palapress	PMMA-base+BM/Palapress	-12,1500	3,696846	-3,28659	0,0010*	-5,1351	-7,4134	-2,9724
AM-base+B/Palapress	PMMA-base+BM/Palapress	-12,7500	3,696846	-3,44889	0,0006*	-9,7107	-11,5005	-7,1008
AM-base+B-PS/primosplint	AM-base+B/freeform plast	-12,8500	3,696846	-3,47594	0,0005*	-2,4730	-3,9866	-1,0980
AM-base+NC/freeprint splint 2	AM-base+M/Palapress	-13,2472	3,610556	-3,66903	0,0002*	-6,4575	-9,8007	-3,1648
AM-base+NC/freeprint splint 2	AM-base+B/freeform plast	-13,2500	3,696846	-3,58414	0,0003*	-3,4421	-4,9909	-1,4898
AM-base+PS/primosplint	AM-base+B-FB/freeform fixgel	-14,3500	3,696846	-3,88169	0,0001*	-3,1542	-4,8680	-1,6398
AM-base+NC/freeform plast	AM-base+B-PS/primosplint	-14,4500	3,696846	-3,90874	<,0001*	-2,8545	-3,8575	-1,8816
AM-base+B/primosplint	AM-base+B-FB/freeform fixgel	-14,7500	3,696846	-3,98989	<,0001*	-3,1285	-4,9566	-1,7930
AM-base+NC/primosplint	AM-base+B-PS/primosplint	-15,2500	3,696846	-4,12514	<,0001*	-3,4078	-4,6381	-2,0176
AM-base+NC/freeprint splint 2	AM-base+B-FB/freeform fixgel	-16,0500	3,696846	-4,34154	<,0001*	-5,2559	-7,1914	-3,2824
AM-base+B-PS/primosplint	AM-base+B-FB/freeform fixgel	-16,2500	3,696846	-4,39564	<,0001*	-4,2412	-6,1011	-2,6034
AM-base+NC/freeform plast	AM-base+M/Palapress	-16,9417	3,610556	-4,69226	<,0001*	-8,3018	-11,2487	-5,3229

Comparison of groups with wet storage (Table S4)		Score Mean Difference	Std Err Dif	Z	p-Value	Hodges-Lehmann	Lower CL	Upper CL
AM-base+NC/freeform fixgel	AM-base+FB/freeform fixgel	-17,2500	3,696846	-4,66614	<,0001*	-6,0985	-8,3402	-4,3822
AM-base+B-FB/freeform plast	PMMA-base+BM/Palapress	-17,4500	3,696846	-4,72024	<,0001*	-8,6830	-10,8906	-6,2316
AM-base+NC/primosplint	AM-base+M/Palapress	-17,6806	3,610556	-4,89691	<,0001*	-8,7887	-12,0533	-6,0347
AM-base+NC/freeform fixgel	AM-base+B-FB/freeform plast	-17,9500	3,696846	-4,85549	<,0001*	-6,7896	-9,3213	-4,4295
AM-base+NC/freeform fixgel	AM-base+B/freeform fixgel	-18,1500	3,696846	-4,90959	<,0001*	-5,6312	-7,4990	-4,0624
AM-base+NC/freeform fixgel	AM-base+B/Palapress	-18,1500	3,696846	-4,90959	<,0001*	-5,4196	-8,8179	-3,8742
AM-base+M/Palapress	PMMA-base+BM/Palapress	-18,3139	3,610556	-5,07232	<,0001*	-11,8228	-14,8649	-8,2936
AM-base+NC/primosplint	AM-base+NC/freeform fixgel	-18,3500	3,696846	-4,96369	<,0001*	-5,4000	-6,8439	-3,6748
AM-base+B/freeprint splint 2	AM-base+B-FB/freeform plast	-18,4500	3,696846	-4,99074	<,0001*	-6,4791	-8,7695	-4,3453
AM-base+NC/primosplint	AM-base+B/primosplint	-18,4500	3,696846	-4,99074	<,0001*	-4,2446	-5,5003	-3,2498
AM-base+B/primosplint	AM-base+FB/freeform fixgel	-18,8500	3,696846	-5,09894	<,0001*	-7,1219	-9,3865	-5,1902
AM-base+NC/primosplint	AM-base+FB/freeform plast	-18,8500	3,696846	-5,09894	<,0001*	-4,4449	-5,8942	-3,4676
AM-base+NC/primosplint	AM-base+PS/primosplint	-18,8500	3,696846	-5,09894	<,0001*	-4,3277	-5,3121	-3,2730
AM-base+PS/primosplint	AM-base+FB/freeform fixgel	-18,9500	3,696846	-5,12599	<,0001*	-7,2627	-9,2427	-5,0502
AM-base+NC/freeform plast	AM-base+NC/freeform fixgel	-18,9500	3,696846	-5,12599	<,0001*	-5,2239	-6,0642	-3,3439
AM-base+NC/freeprint splint 2	AM-base+FB/freeform fixgel	-19,0500	3,696846	-5,15304	<,0001*	-8,8405	-10,9127	-6,5951
AM-base+B/primosplint	AM-base+B-FB/freeform plast	-19,1500	3,696846	-5,18009	<,0001*	-7,4960	-9,9939	-5,4425
AM-base+PS/primosplint	AM-base+B-FB/freeform plast	-19,2500	3,696846	-5,20714	<,0001*	-7,4853	-9,9583	-5,6113
AM-base+B-PS/primosplint	AM-base+FB/freeform fixgel	-19,2500	3,696846	-5,20714	<,0001*	-8,2838	-10,0989	-5,9878
AM-base+NC/freeprint splint 2	AM-base+B/freeform fixgel	-19,2500	3,696846	-5,20714	<,0001*	-8,3885	-10,4596	-6,5422
AM-base+NC/freeprint splint 2	AM-base+B/Palapress	-19,2500	3,696846	-5,20714	<,0001*	-8,6215	-11,5023	-6,4862
AM-base+NC/freeprint splint 2	AM-base+B-FB/freeform plast	-19,3500	3,696846	-5,23419	<,0001*	-9,3882	-12,0491	-6,9805
AM-base+NC/primosplint	AM-base+NC/Palapress	-19,4487	3,652685	-5,32449	<,0001*	-20,4958	-22,4972	-18,2266
AM-base+B/freeform fixgel	PMMA-base+BM/Palapress	-19,4500	3,696846	-5,26124	<,0001*	-9,7567	-11,1787	-7,9956
AM-base+PS/primosplint	AM-base+B/freeform fixgel	-19,4500	3,696846	-5,26124	<,0001*	-6,7449	-8,3178	-5,0804
AM-base+NC/freeform plast	AM-base+B/primosplint	-19,4500	3,696846	-5,26124	<,0001*	-3,7682	-4,9305	-2,6182
AM-base+B-PS/primosplint	AM-base+B-FB/freeform plast	-19,5500	3,696846	-5,28829	<,0001*	-8,6973	-11,2173	-6,3782
AM-base+NC/freeform plast	AM-base+FB/freeform plast	-19,5500	3,696846	-5,28829	<,0001*	-3,9840	-5,1593	-2,8172
AM-base+FB/freeform fixgel	PMMA-base+BM/Palapress	-19,6500	3,696846	-5,31534	<,0001*	-9,1059	-11,3156	-7,3201
AM-base+B/primosplint	AM-base+B/freeform fixgel	-19,6500	3,696846	-5,31534	<,0001*	-6,6907	-8,2713	-5,0316
AM-base+B-PS/primosplint	AM-base+B/freeform fixgel	-19,7500	3,696846	-5,34239	<,0001*	-7,7014	-9,3246	-6,0785
AM-base+NC/freeform plast	AM-base+PS/primosplint	-19,7500	3,696846	-5,34239	<,0001*	-3,6395	-4,5830	-2,7236
AM-base+NC/primosplint	AM-base+B/freeprint splint 2	-19,7500	3,696846	-5,34239	<,0001*	-5,4508	-6,5636	-4,5557
AM-base+NC/primosplint	AM-base+B/freeform plast	-19,8500	3,696846	-5,36944	<,0001*	-5,7443	-7,0582	-4,7319
AM-base+B/freeform plast	PMMA-base+BM/Palapress	-19,9500	3,696846	-5,39649	<,0001*	-14,8561	-16,3218	-13,4641
AM-base+FB/freeform plast	PMMA-base+BM/Palapress	-19,9500	3,696846	-5,39649	<,0001*	-16,0274	-17,6152	-14,6463
AM-base+B/freeprint splint 2	PMMA-base+BM/Palapress	-19,9500	3,696846	-5,39649	<,0001*	-15,2130	-16,5071	-14,2435
AM-base+B-FB/freeform fixgel	PMMA-base+BM/Palapress	-19,9500	3,696846	-5,39649	<,0001*	-13,1111	-14,8396	-11,3273

Comparison of groups with wet storage (Table S4)		Score Mean Difference	Std Err Dif	Z	p-Value	Hodges-Lehmann	Lower CL	Upper CL
AM-base+PS/primosplint	PMMA-base+BM/Palapress	-19,9500	3,696846	-5,39649	<,0001*	-16,5252	-17,7564	-15,0420
AM-base+B/primosplint	PMMA-base+BM/Palapress	-19,9500	3,696846	-5,39649	<,0001*	-16,3040	-17,8118	-15,1960
AM-base+B-PS/primosplint	PMMA-base+BM/Palapress	-19,9500	3,696846	-5,39649	<,0001*	-17,3826	-18,7205	-16,0075
AM-base+NC/freeform fixgel	PMMA-base+BM/Palapress	-19,9500	3,696846	-5,39649	<,0001*	-15,2437	-16,8393	-14,1040
AM-base+NC/freeform fixgel	AM-base+BM/Palapress	-19,9500	3,696846	-5,39649	<,0001*	-10,2872	-13,0078	-7,7612
AM-base+NC/freeform plast	PMMA-base+BM/Palapress	-19,9500	3,696846	-5,39649	<,0001*	-20,3019	-21,1516	-19,4259
AM-base+NC/freeform plast	AM-base+B/freeform plast	-19,9500	3,696846	-5,39649	<,0001*	-5,0826	-6,3100	-4,1117
AM-base+NC/freeform plast	AM-base+B-FB/freeform plast	-19,9500	3,696846	-5,39649	<,0001*	-11,3246	-13,2302	-9,3616
AM-base+NC/freeform plast	AM-base+B/freeprint splint 2	-19,9500	3,696846	-5,39649	<,0001*	-5,0386	-5,7142	-4,1776
AM-base+NC/freeform plast	AM-base+FB/freeform fixgel	-19,9500	3,696846	-5,39649	<,0001*	-11,4332	-12,5788	-8,5434
AM-base+NC/freeform plast	AM-base+B/freeform fixgel	-19,9500	3,696846	-5,39649	<,0001*	-10,5157	-11,7773	-9,1099
AM-base+NC/freeform plast	AM-base+B-FB/freeform fixgel	-19,9500	3,696846	-5,39649	<,0001*	-6,8542	-8,2843	-5,4292
AM-base+NC/freeform plast	AM-base+B/Palapress	-19,9500	3,696846	-5,39649	<,0001*	-10,2759	-11,5291	-8,8156
AM-base+NC/freeform plast	AM-base+BM/Palapress	-19,9500	3,696846	-5,39649	<,0001*	-14,4813	-16,6215	-12,8320
AM-base+NC/freeprint splint 2	PMMA-base+BM/Palapress	-19,9500	3,696846	-5,39649	<,0001*	-18,1477	-19,9559	-16,5825
AM-base+NC/freeprint splint 2	AM-base+BM/Palapress	-19,9500	3,696846	-5,39649	<,0001*	-13,1067	-15,6039	-10,4922
AM-base+NC/primosplint	PMMA-base+BM/Palapress	-19,9500	3,696846	-5,39649	<,0001*	-20,8838	-21,9776	-19,4773
AM-base+NC/primosplint	AM-base+B-FB/freeform plast	-19,9500	3,696846	-5,39649	<,0001*	-11,7503	-14,1717	-9,7395
AM-base+NC/primosplint	AM-base+FB/freeform fixgel	-19,9500	3,696846	-5,39649	<,0001*	-11,6673	-13,4455	-9,1721
AM-base+NC/primosplint	AM-base+B/freeform fixgel	-19,9500	3,696846	-5,39649	<,0001*	-11,0565	-12,4358	-9,3088
AM-base+NC/primosplint	AM-base+B-FB/freeform fixgel	-19,9500	3,696846	-5,39649	<,0001*	-7,4270	-9,0537	-6,0722
AM-base+NC/primosplint	AM-base+B/Palapress	-19,9500	3,696846	-5,39649	<,0001*	-10,7671	-12,6525	-9,3030
AM-base+NC/primosplint	AM-base+BM/Palapress	-19,9500	3,696846	-5,39649	<,0001*	-15,1385	-17,6128	-13,4641

Table S5 Failure mode and measured Shear Bond Strength in Failure Groups per Bonding interface and repair material

Bonding interface and repair material	Storage (ageing)	Failure Mode								
		adhesive			cohesive			mixed		
		SBS(MPa)			SBS(MPa)			SBS(MPa)		
		N	Mean	Std Dev	N	Mean	Std Dev	N	Mean	Std Dev
PMMA-base+BM/Palapress	dry storage	0	n.a.	n.a.	20	25.18	2.59	0	n.a.	n.a.
	wet storage	0	n.a.	n.a.	20	23.57	2	0	n.a.	n.a.
AM-base+B/freeform plast	dry storage	7	4.1	1.08	8	9.52	2.01	5	6.5	1.76
	wet storage	0	n.a.	n.a.	14	9.62	1.78	6	7.07	0.9
AM-base+FB/freeform plast	wet storage	5	5.21	0.46	4	10.72	1.84	11	7.75	1.28
AM-base+B-FB/freeform plast	wet storage	0	n.a.	n.a.	20	15.32	4.39	0	n.a.	n.a.
AM-base+B/freeprint splint	dry storage	0	n.a.	n.a.	20	15.76	3.94	0	n.a.	n.a.
	wet storage	0	n.a.	n.a.	13	9.1	1.08	7	7.02	0.95
AM-base+FB/freeform fixgel	wet storage	0	n.a.	n.a.	18	14.94	3.19	2	8.67	1.74
AM-base+B/freeform fixgel	dry storage	11	7.97	4.65	2	16.61	0.39	7	7.43	3.55
	wet storage	0	n.a.	n.a.	20	14.12	3.21	0	n.a.	n.a.
AM-base+B-FB/freeform fixgel	wet storage	0	n.a.	n.a.	19	11.14	3.2	1	6.88	n.a.
AM-base+PS/primosplint	dry storage	0	n.a.	n.a.	12	6.96	3.92	8	3.86	0.84
	wet storage	0	n.a.	n.a.	12	8.1	1.42	8	5.98	0.88
AM-base+B/primosplint	dry storage	10	2.13	1.22	6	6.28	1.56	4	5.29	2.04
	wet storage	1	3.98	n.a.	0	n.a.	n.a.	19	7.39	1.56
AM-base+B-PS/primosplint	dry storage	2	3.16	1.05	11	8.29	2.53	7	5.29	1.61
	wet storage	1	1.3	n.a.	9	7.92	1.39	10	5.14	1.4
AM-base+M/Palapress	dry storage	0	n.a.	n.a.	20	19.56	4.19	0	n.a.	n.a.
	wet storage	2	3.88	1.66	12	14.97	4.35	4	8.01	1.24
AM-base+B/Palapress	dry storage	0	n.a.	n.a.	20	17.56	3.02	0	n.a.	n.a.
	wet storage	0	n.a.	n.a.	20	16.08	6.91	0	n.a.	n.a.

Table S5 Failure mode and measured Shear Bond Strength in Failure Groups per Bonding interface and repair material

Bonding interface and repair material	Storage (ageing)	Failure Mode								
		adhesive			cohesive			mixed		
		SBS(MPa)								
		N	Mean	Std Dev	N	Mean	Std Dev	N	Mean	Std Dev
AM-base+BM/Palapress	dry storage	0	n.a.	n.a.	20	22.7	2.47	0	n.a.	n.a.
	wet storage	0	n.a.	n.a.	20	19.18	4.72	0	n.a.	n.a.
AM-base+NC/freeform fixgel	dry storage	10	3.6	2.01	6	7.45	0.94	1	3.19	n.a.
	wet storage	13	7.26	2.37	2	10.47	2.21	5	9.38	1.11
AM-base+NC/freeform plast	dry storage	15	3.75	2.47	2	8.48	1.24	3	5.27	2.19
	wet storage	19	3.4	0.74	0	n.a.	n.a.	1	5.14	n.a.
AM-base+NC/freeprint splint	dry storage	0	n.a.	n.a.	20	13.99	3.31	0	n.a.	n.a.
	wet storage	6	2.61	1.15	5	9.19	1.53	9	5.52	1.5
AM-base+NC/Palapress	dry storage	0	n.a.	n.a.	20	21.41	4.06	0	n.a.	n.a.
	wet storage	0	n.a.	n.a.	19	22.97	5.42	0	n.a.	n.a.
AM-base+NC/primosplint	dry storage	11	3.14	1.84	9	4.87	1.76	0	n.a.	n.a.
	wet storage	18	2.71	1.27	0	n.a.	n.a.	2	5.22	1.26
