# Supplementary material for: Bonding Interface and Repairability of 3D-Printed Intraoral Splints: Shear Bond Strength to Current Polymers, with and without Ageing
Source: Materials (Basel). 2021 Jul 14;14(14):3935. doi: 10.3390/ma14143935 (PMC8307865; doi:10.3390/ma14143935)

Figure S1: Schematic drawing of shear bond testing setup

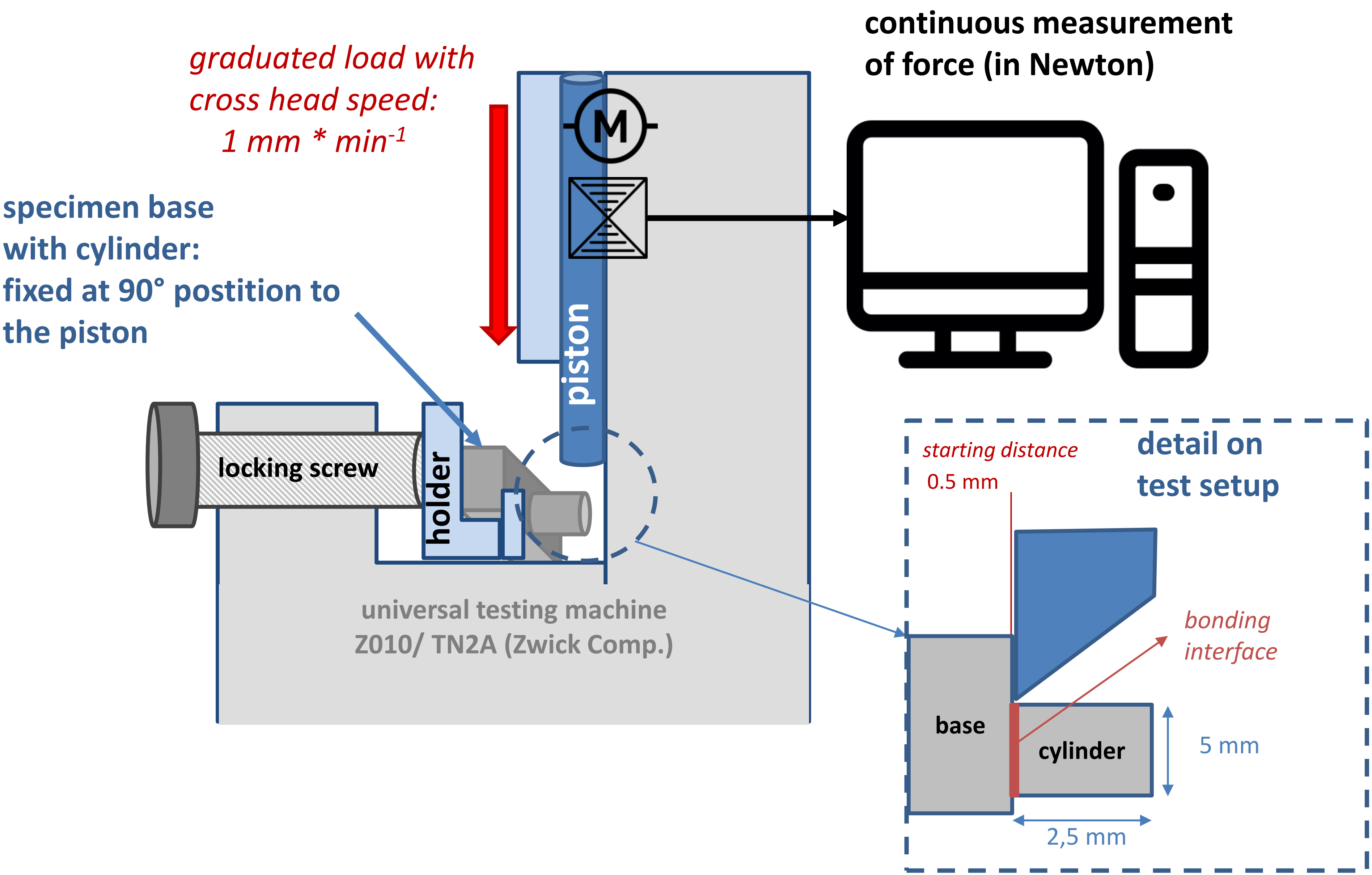

Kuscu et al. 2021 – supplementary material

PMMA base+BM: SEM (1000x); DRY STORAGE

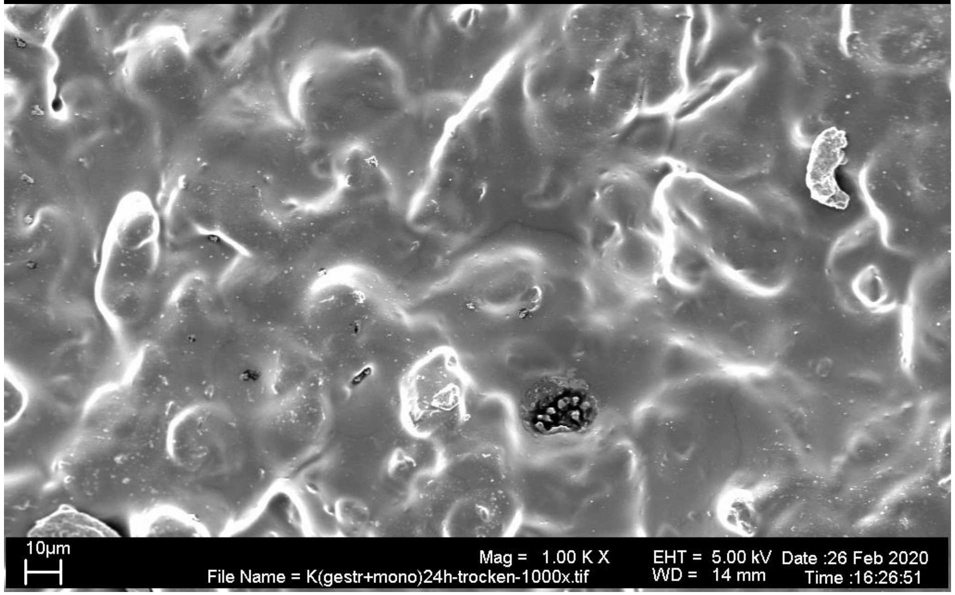

PMMA base+BM: SEM (1000x), WET STORAGE

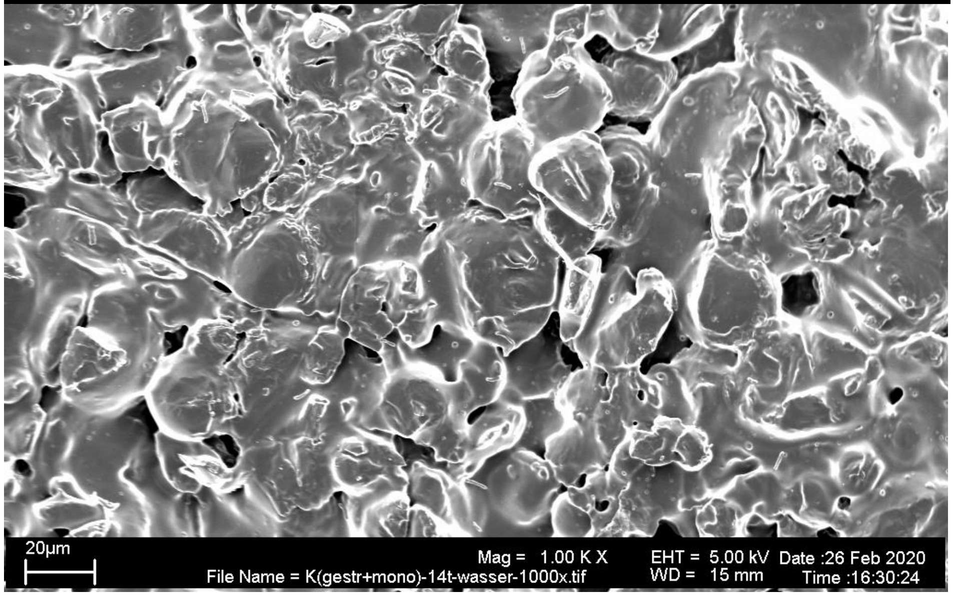

PMMA base+BM: DRY STORAGE; 3D view of surface (Ra)

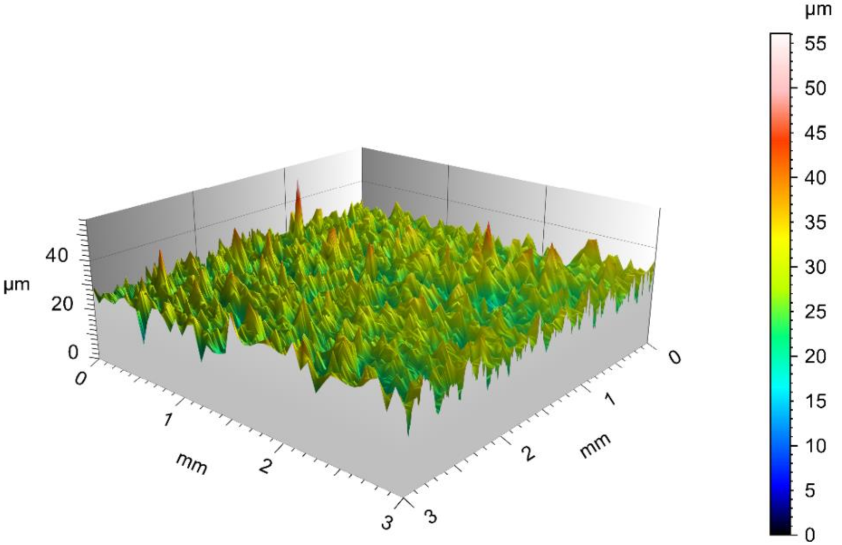

PMMA base+BM: WET STORAGE; 3D view of surface (Ra)

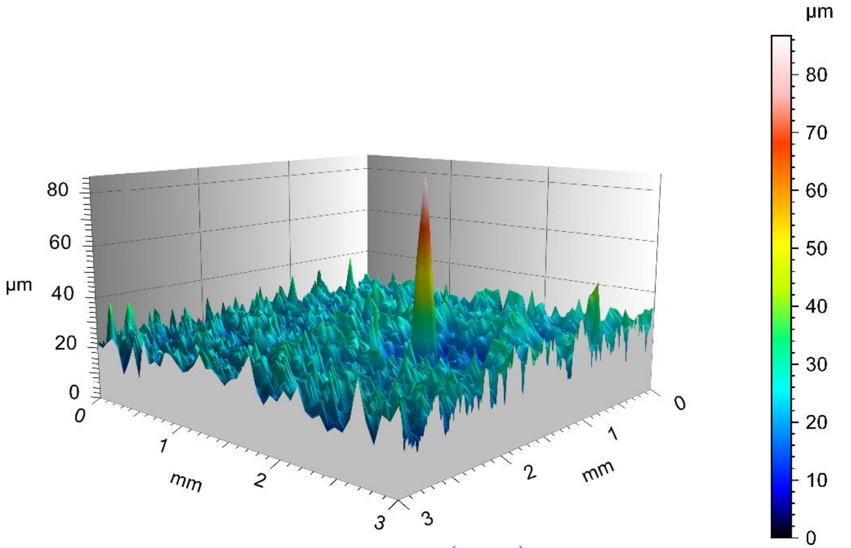

AM base+NC: SEM (1000x); DRY STORAGE

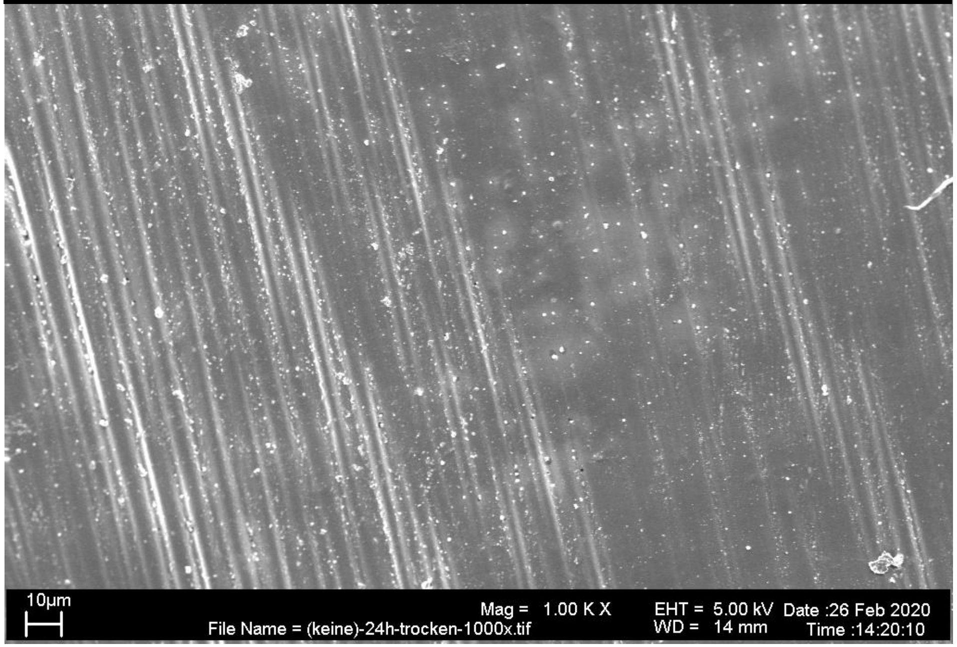

AM base+NC: SEM (1000x); WET STORAGE

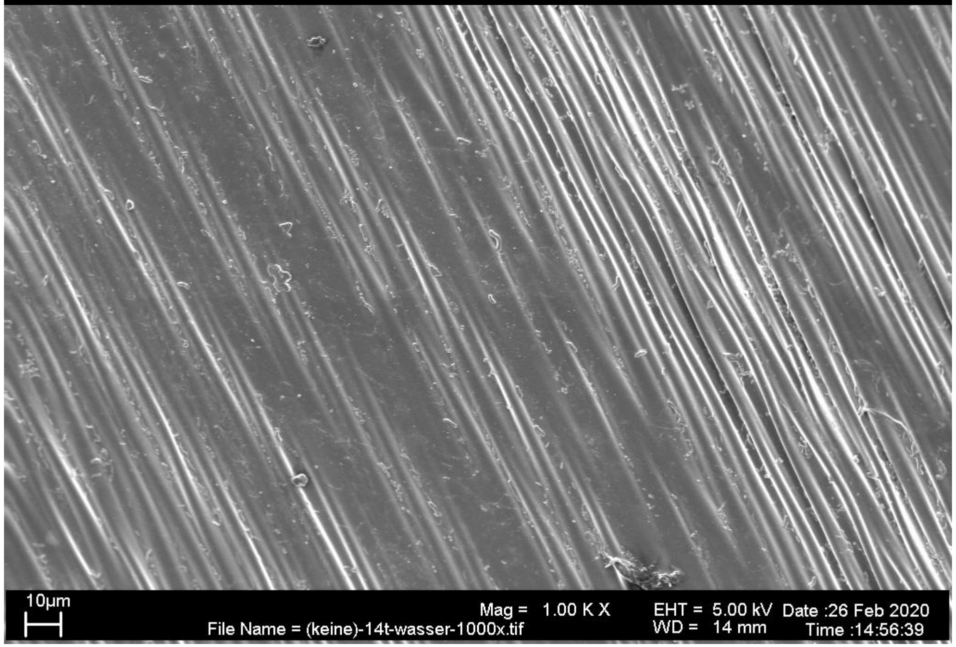

AM base+NC: DRY STORAGE; 3D view of surface (Ra)

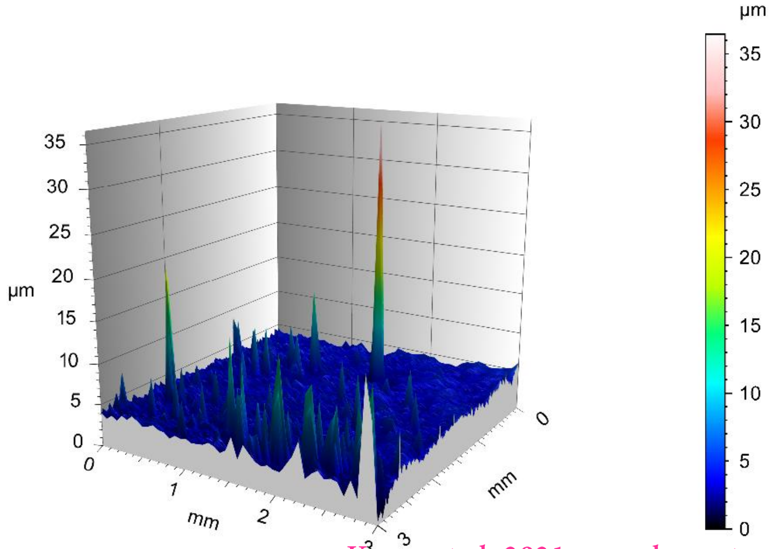

AM base+NC: WET STORAGE; 3D view of surface (Ra)

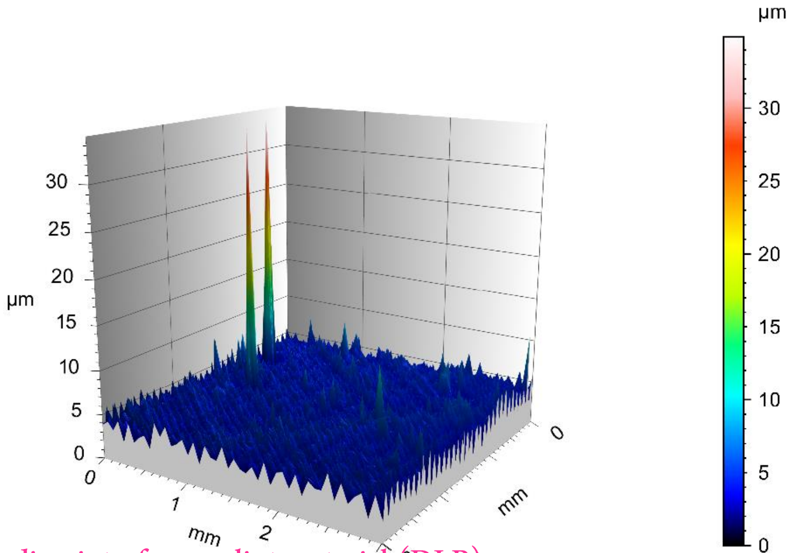

AM base+B: SEM (1000x); DRY STORAGE

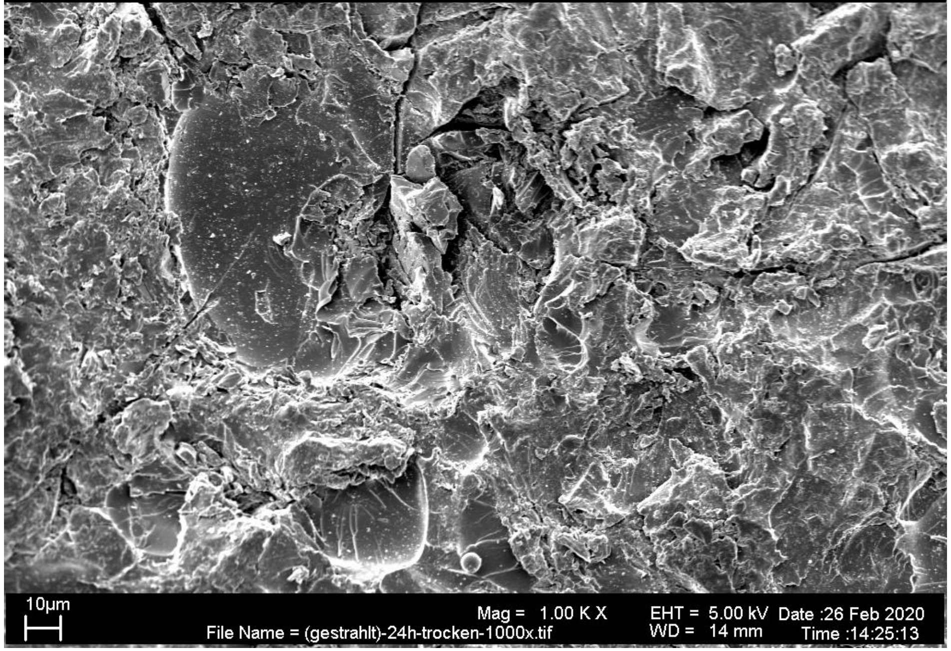

AM base+B: SEM (1000x); WET STORAGE

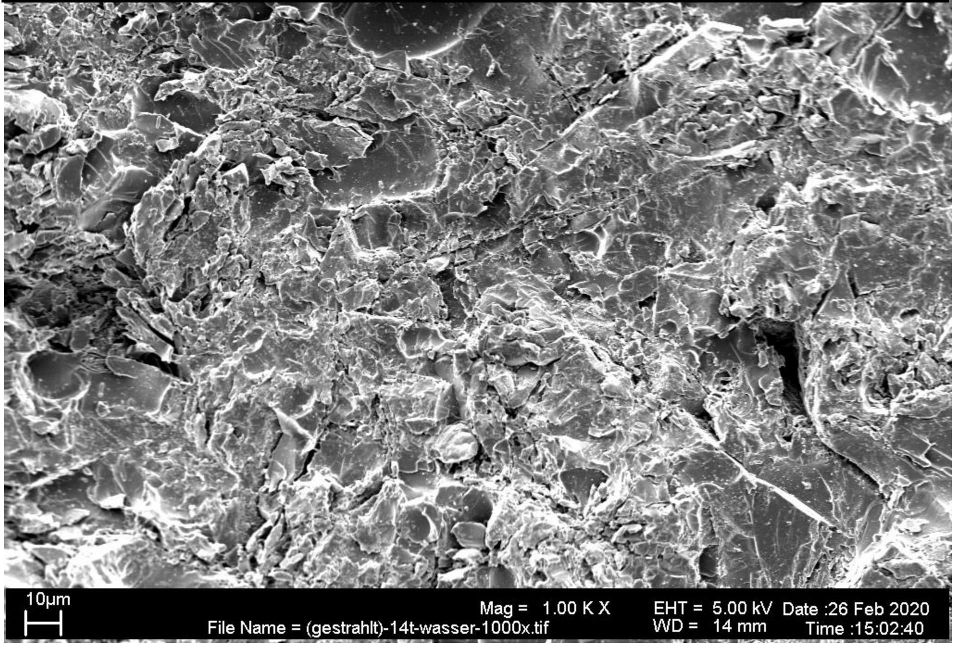

AM base+B: DRY STORAGE; 3D view of surface (Ra)

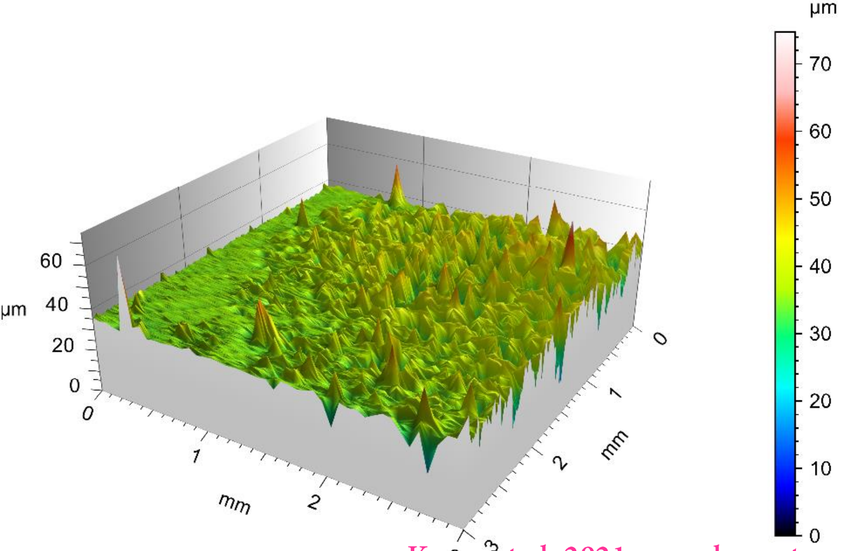

AM base+B: WET STORAGE; 3D view of surface (Ra)

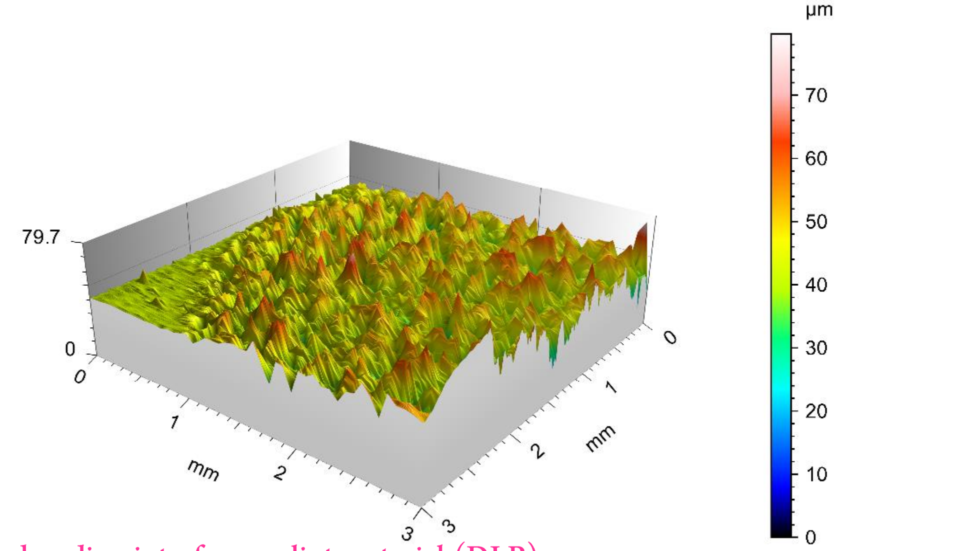

AM base+M: SEM (1000x); DRY STORAGE

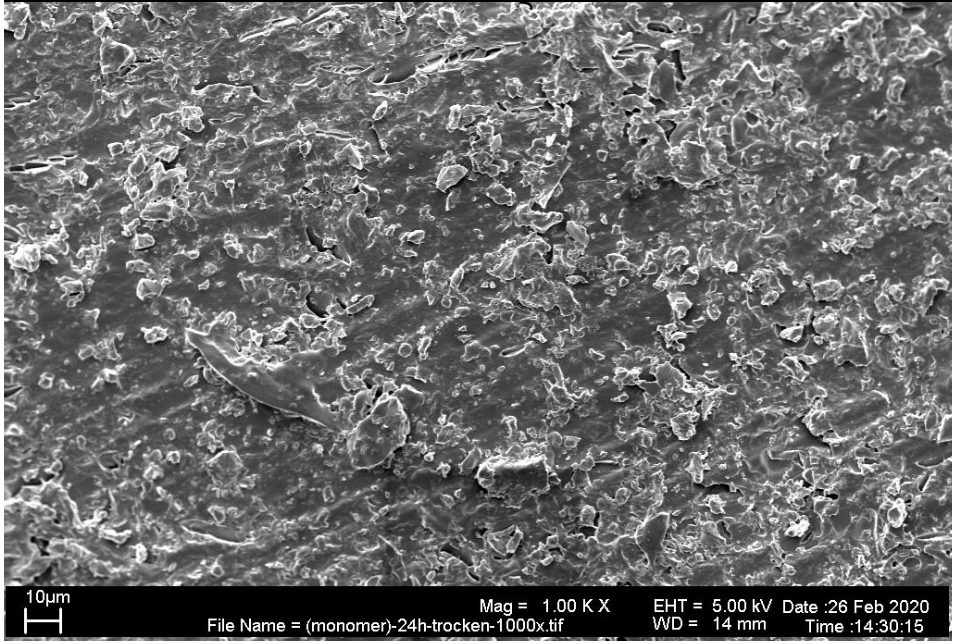

AM base+M: SEM (1000x); WET STORAGE

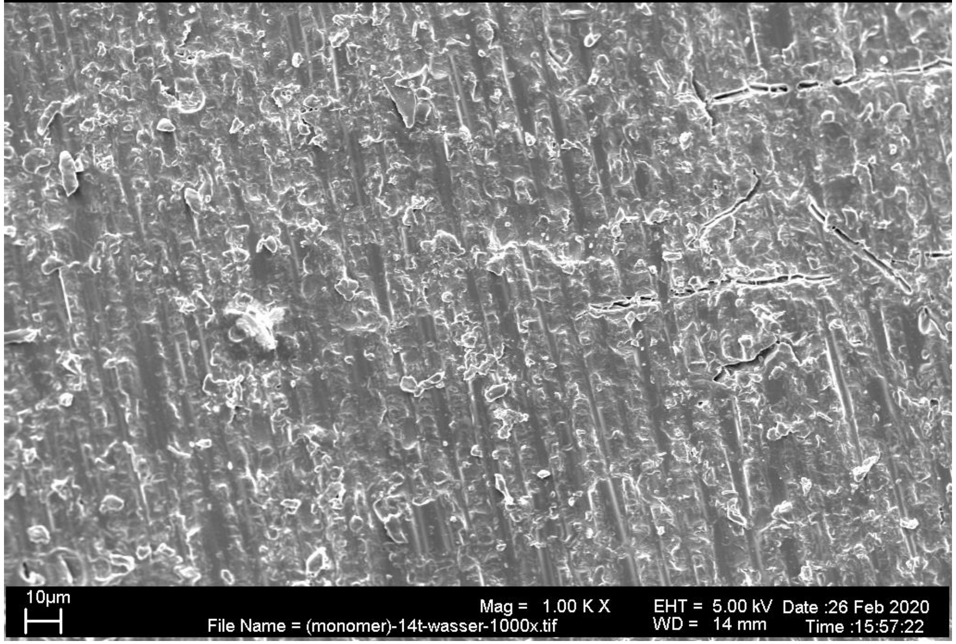

AM base+M: DRY STORAGE; 3D view of surface (Ra)

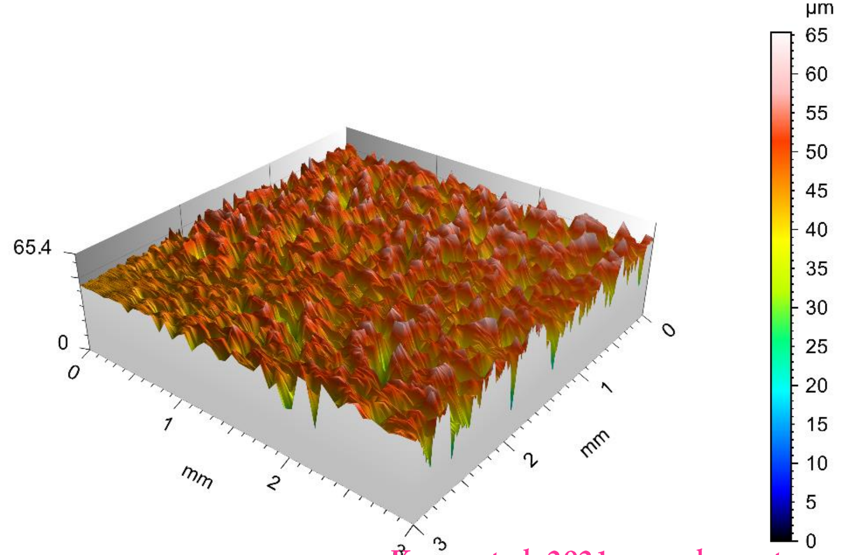

AM base+M: WET STORAGE; 3D view of surface (Ra)

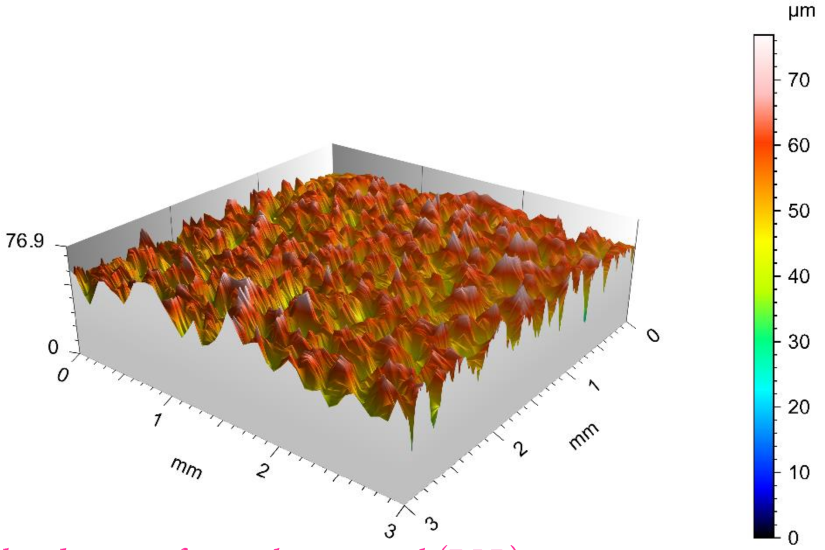

AM base+BM: SEM (1000x); DRY STORAGE

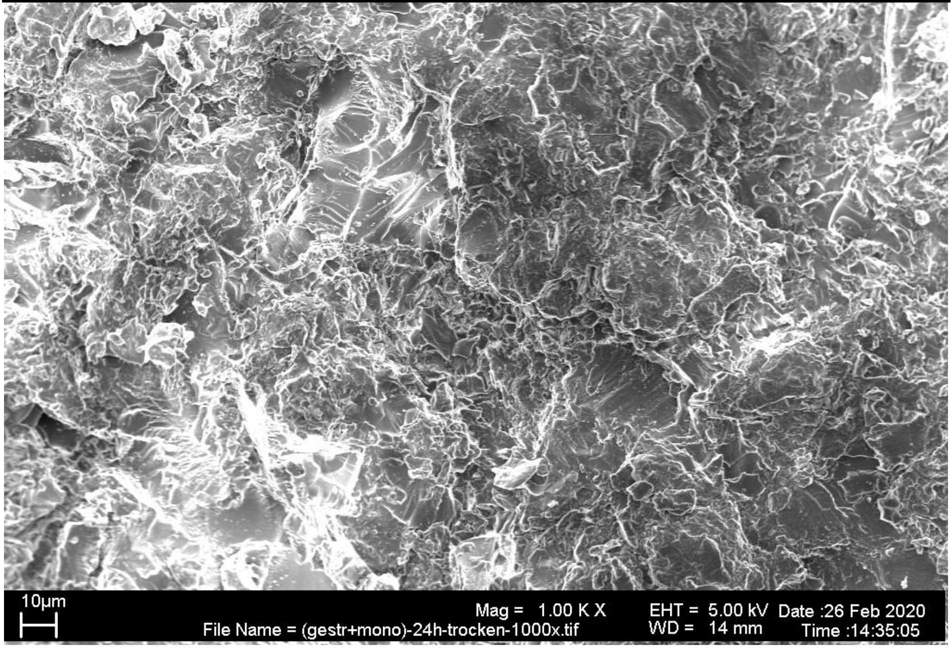

AM base+BM: SEM (1000x); WET STORAGE

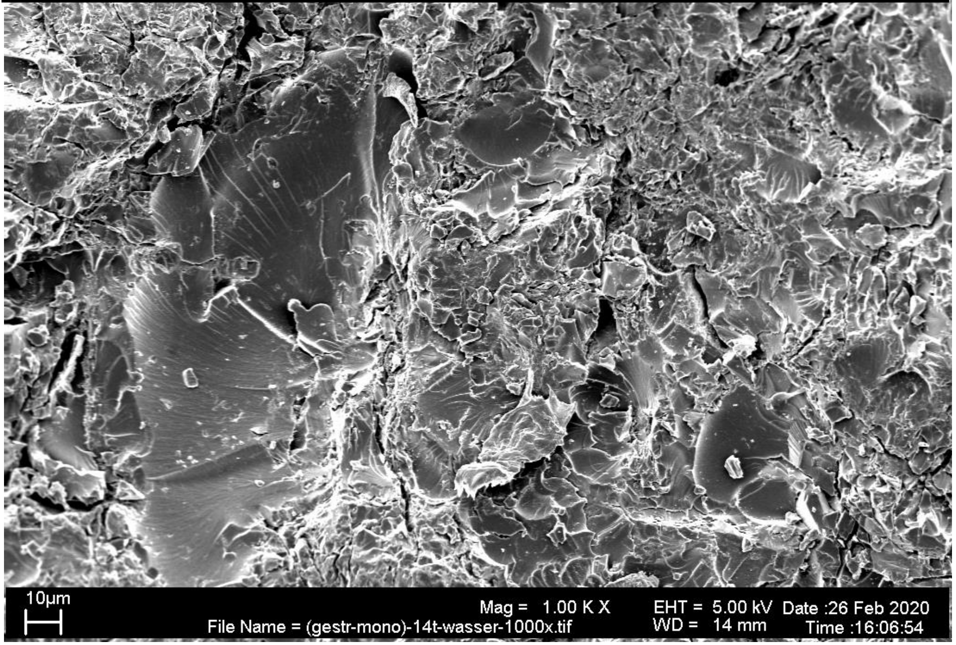

AM base+BM: DRY STORAGE; 3D view of surface (Ra)

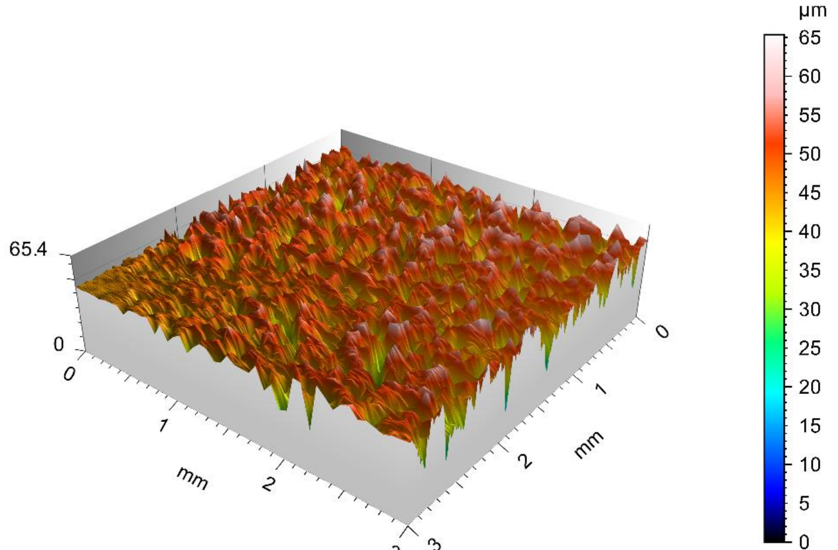

AM base+BM: WET STORAGE; 3D view of surface (Ra)

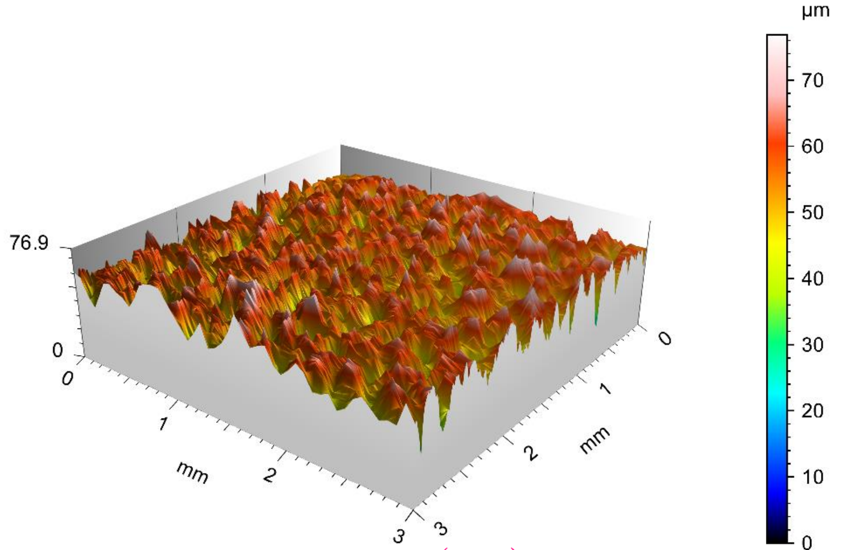

AM base+PS: SEM (1000x); DRY STORAGE

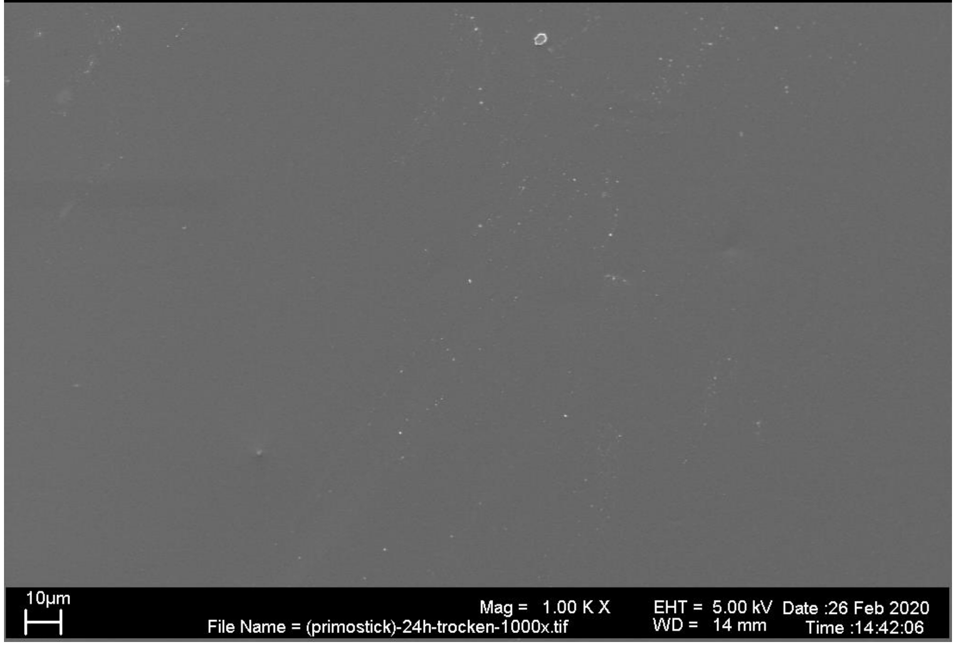

AM base+PS: SEM (1000x); WET STORAGE

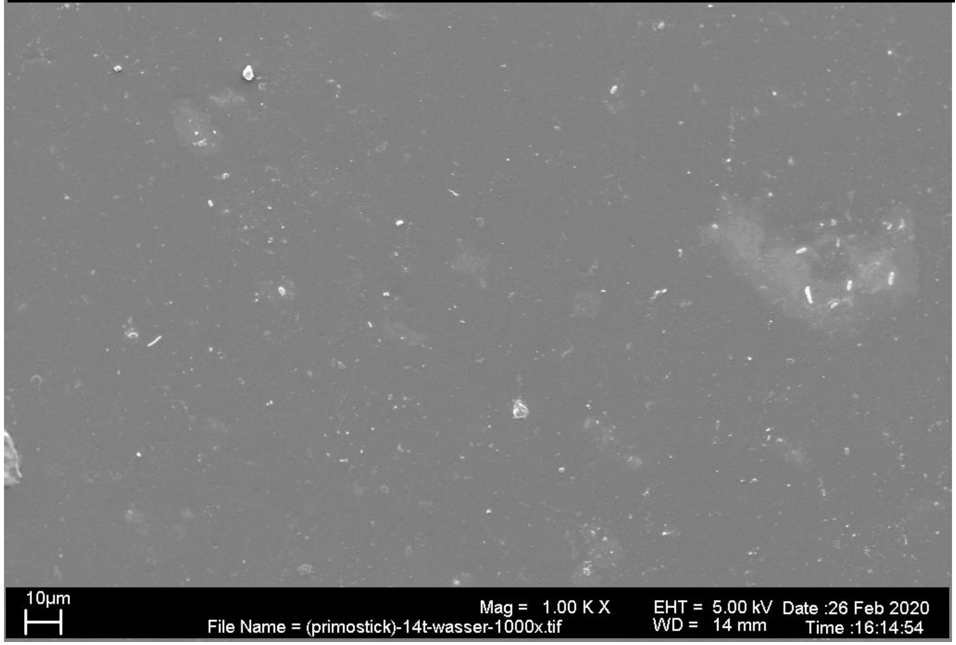

AM base+PS: DRY STORAGE; 3D view of surface (Ra)

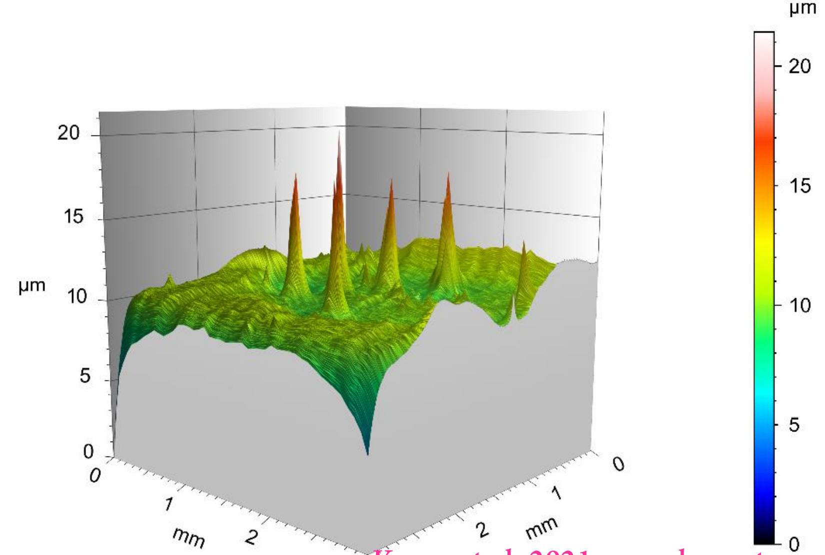

AM base+PS: WET STORAGE; 3D view of surface (Ra)

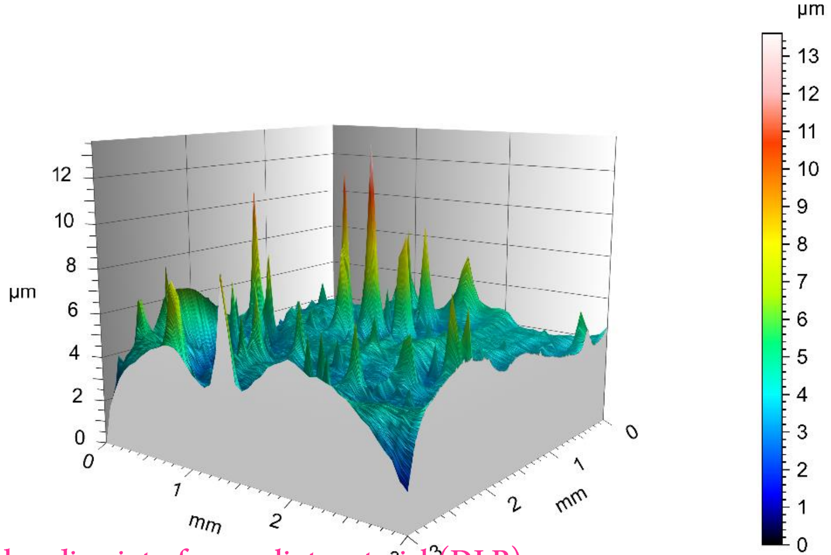

AM base+B-PS: SEM (1000x); DRY STORAGE

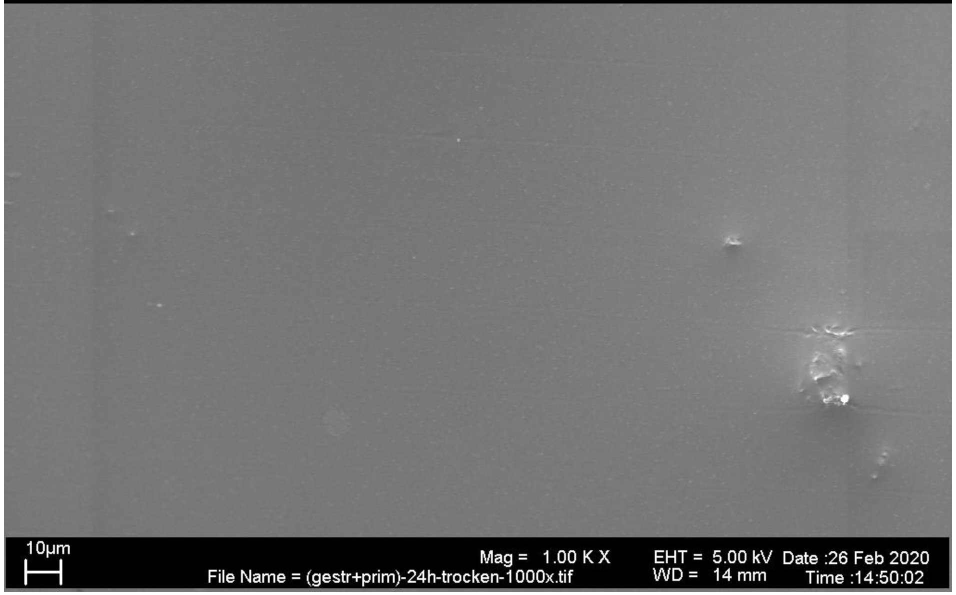

AM base+B-PS: SEM (1000x); WET STORAGE

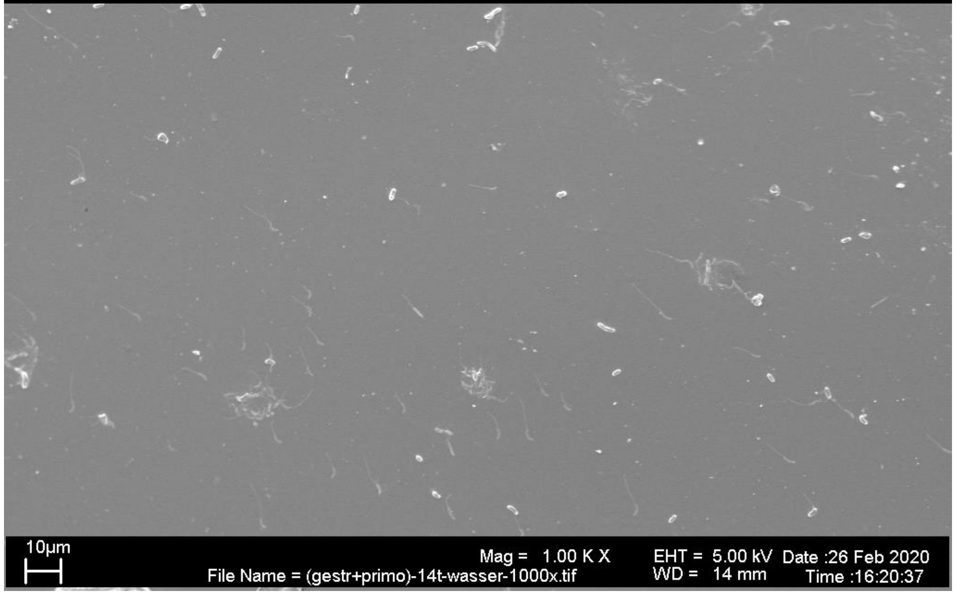

AM base+B-PS: DRY STORAGE; 3D view of surface (Ra)

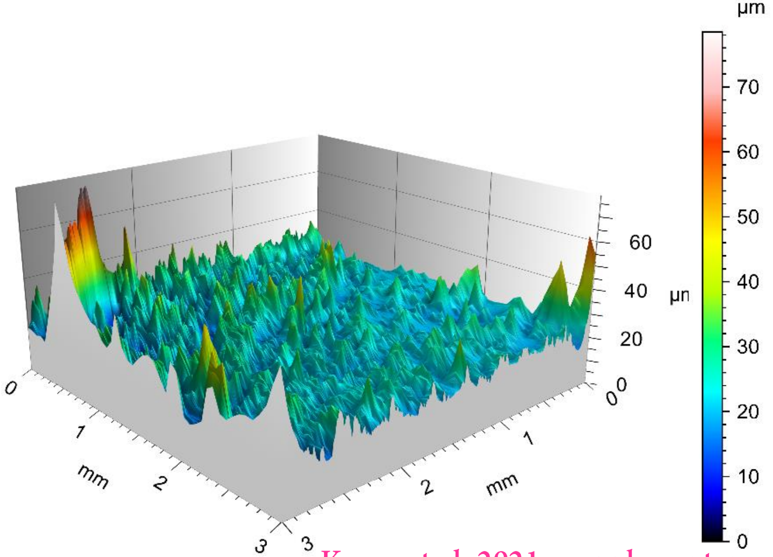

AM base+B-PS: WET STORAGE; 3D view of surface (Ra)

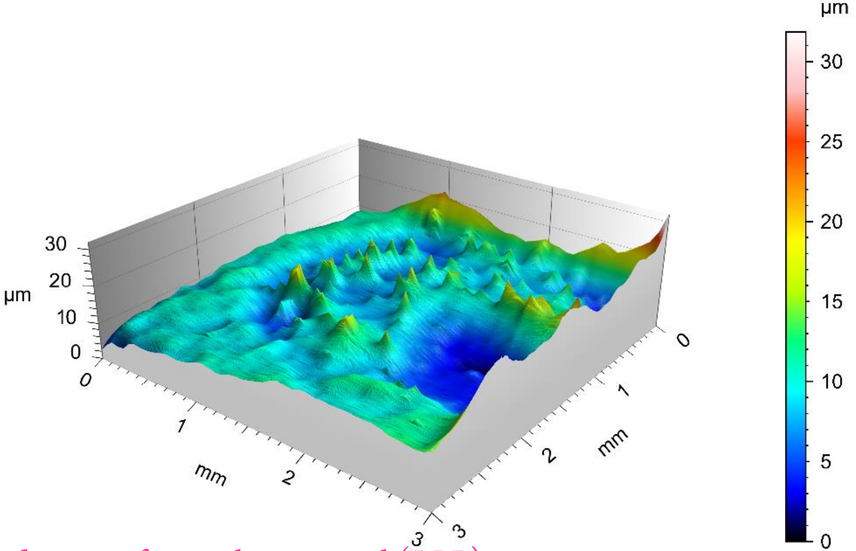

AM base+FB: SEM (1000x); WET STORAGE

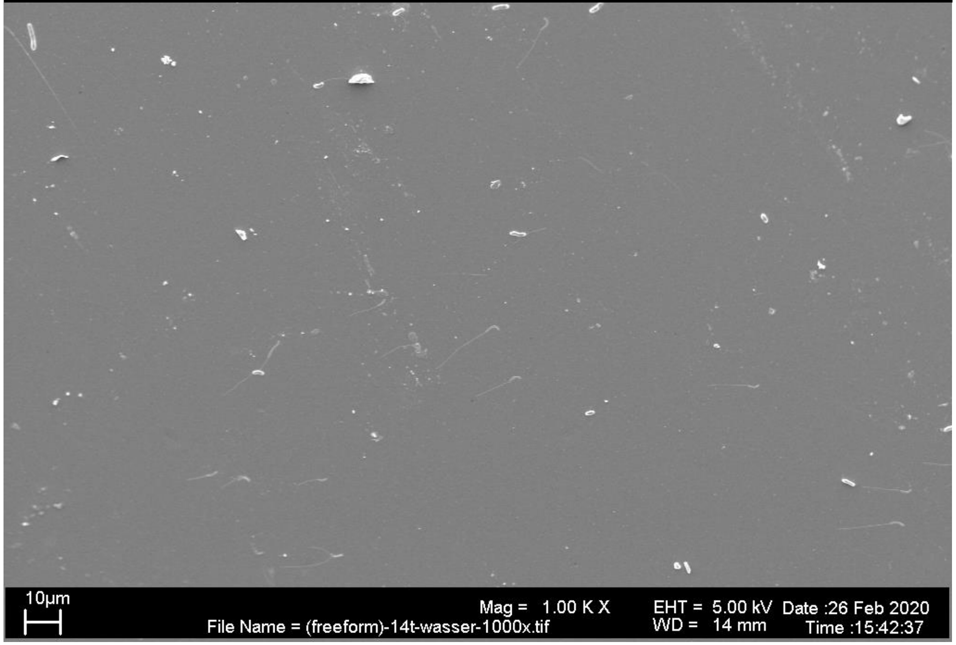

AM base+B-FB: SEM(1000x);WET STORAGE

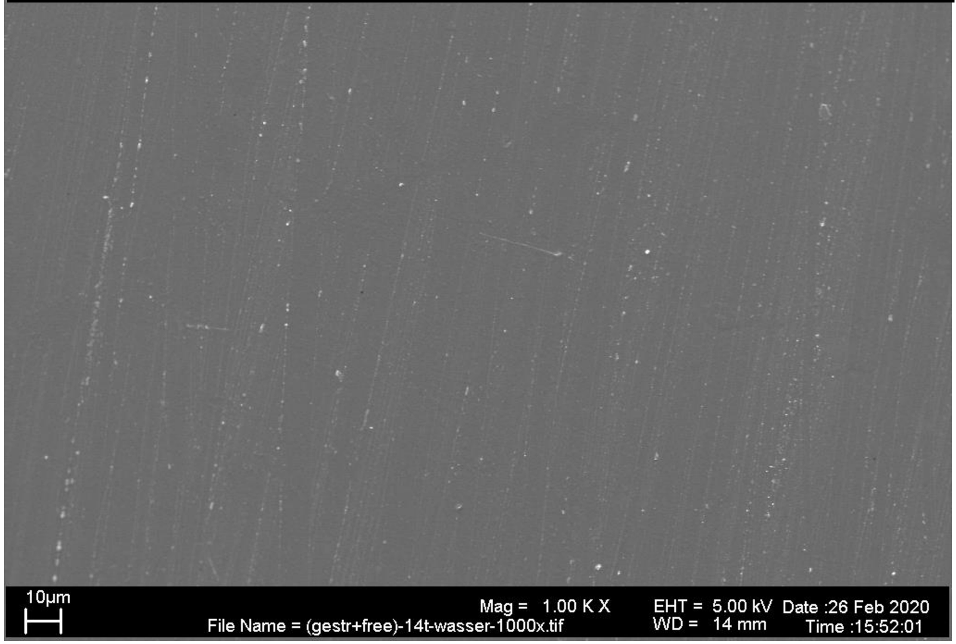

AM base+FB: WET STORAGE; 3D view of surface (Ra)

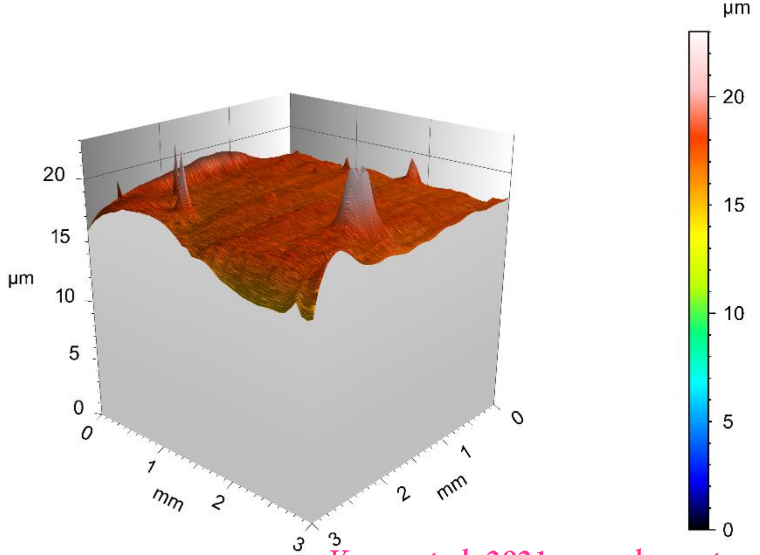

AM base+B-FB: WET STORAGE; 3D view of surface (Ra)

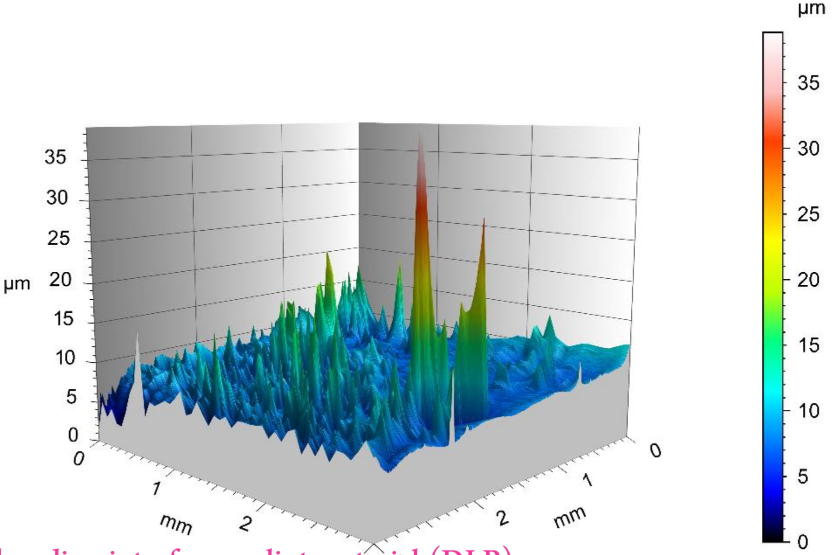

Supplement: Supplementary file 1 [file materials-14-03935-s001.zip › Supplement_2_Figures_S1-S9.pdf]
